# Co-ADAM: a co-evolutionary signaling game framework for equilibrium cyber deception in industrial IoT

**DOI:** 10.1038/s41598-026-60920-0

**Published:** 2026-07-06

**Authors:** Usman Wushishi, Nasir Hussain, Altaf Hussain, Amal Al-Rasheed, Zahid Ullah

**Affiliations:** 1 School of Computer Science and Technology, Chongqing University of Posts and Telecommunications, Chongqing, 400065 China; 2 Department of Information Systems, College of Computer and Information Sciences, Princess Nourah Bint Abdulrahman University, P.O. Box 84428, 11671 Riyadh, Saudi Arabia; 3 Information Systems Department, College of Computer and Information Sciences, Imam Mohammad Ibn Saud Islamic University (IMSIU), 11432 Riyadh, Saudi Arabia

**Keywords:** Industrial internet of things, Cyber deception, Co-evolutionary reinforcement learning, Signaling game, Deep Q-network, Nash equilibrium, Adversarial skepticism, Attack mitigation

## Abstract

Cyber deception in Industrial Internet of Things (IIoT) environments faces a fundamental challenge, insufficient deception investment fails to trap attackers, while excessive deployment causes rational adversaries to accumulate skepticism, progressively neutralizing deceptive mechanisms. To the best of our knowledge, no existing reinforcement learning (RL) framework has jointly characterized the optimal deception investment level under these dynamics and trained against a co-evolutionary adversary that explicitly models skepticism accumulation. This paper introduces Co-ADAM, a Co-evolutionary Adaptive Deception-Aware Multi-mechanism defense framework that formalizes IIoT cyber deception as a constrained Markov decision process (CMDP) with an embedded signaling game. We establish that the co-evolutionary training will reach some equilibrium of skepticism (Lemma 1, Propositions 1, 2) and characterize the resulting joint policy as an *ε*-Nash deception equilibrium with exploitability *ε* = 2.4523 (4.43% normalized). A deep Q-network (DQN) defender is trained against an adaptive attacker pool of size *K* = 10 across 5000 episodes, with skepticism dynamics, attacker momentum, and budget constraints embedded directly in the environment. Compared to seven baselines over three IIoT datasets, CIC-IIoT 2025, WUSTL-IIoT 2021 and Edge-IIoTset, Co-ADAM attains an Attack Mitigation Rate (AMR) of 0.8363 under random and 0.8033 under adaptive attackers, with cumulative rewards of 147.91 and 102.52 respectively, all differences are statistically significant at *p* < 0.001. The learned policy transfers across heterogeneous IIoT environments with 99.61% mean AMR retention, scales to 1000 devices with less than 0.26% AMR degradation, and converges empirically at episode 1377. The most significant element of the architecture confirmed in ablation as adversarial skepticism (Cohen’s *d* = 3.57), forms the foundation of principled skepticism modeling as a critical component of deception-based IIoT defense. Physical validation on a 10-day IIoT testbed comprising a Centerm D660 honeypot, three ESP32 sensors, and a Kali Linux attack machine confirms simulation findings with testbed AMR of 0.9975 exceeding simulation AMR of 0.8363.

## Introduction

Due to the spread of Industrial Internet of Things (IIoT) devices, the attack surface of critical infrastructure has essentially increased. Unlike conventional IT networks, IIoT environments operate under strict latency, energy, and computational constraints that preclude the deployment of heavyweight intrusion detection or cryptographic solutions^1–3^. Cyber deception, the deliberate deployment of honeypots, decoy documents, and false network signals to mislead and trap adversaries has emerged as a computationally lightweight and strategically potent complement to conventional defenses^4–6^.

However, current systems of Deception are based on an important and yet largely untestable supposition, which is that attackers will remain naive when it comes to deceptive stimuli in repeated interaction. Practically, a rational opponent, who is waging a prolonged attack on an IIoT network, does not stay in one place. Through repeated encounters with deceptive mechanisms, an attacker accumulates skepticism, a learned disposition to disbelieve or evade cues that in the past have caused honeypot engagement instead of successful attack. This creates a fundamental deception dilemma that no existing framework has explicitly defined, a defender who invests too little in deception fails to trap attackers, while one who invests too much causes skepticism to accumulate, progressively neutralizing the deceptive mechanisms that constitute the defense.

The optimal deception investment level is therefore neither zero nor maximal, it is an equilibrium quantity that depends on the joint dynamics of defender strategy and attacker adaptation. Current reinforcement learning (RL) solutions to IIoT defense either neglect deception or assume that it is a fixed auxiliary mechanism without modeling attacker responses^7,8^. Game-theoretic formulations using Stackelberg or Nash equilibrium models capture strategic interaction but assume that the types of the attackers are fixed and all know the game structure, both of which are often false in real-life^9–11^.

There is evidence that co-evolutionary training techniques have been proven resilient in the presence of adaptive adversaries in other areas, but have not been applied to deception-aware IIoT defense^12,13^. These limitations result in defense policies that are either too passive to trap attackers or too aggressive to remain effective as attackers adapt, leaving IIoT infrastructure exposed to sophisticated adversaries that learn from repeated deception encounters. The result is a tripartite gap, including limited or no existing work simultaneously provides formal deception equilibrium guarantees, trains against a co-evolutionary adversary with explicit skepticism dynamics, and validates the resulting policy across heterogeneous real-world IIoT threat distributions. This paper closes that gap with four contributions:

- We formalize IIoT cyber deception as a (CMDP) with an embedded signaling game in which the defender’s deception investment dynamically modulates attacker skepticism, which in turn affects attack success probability. Analytically we deduce conditions of a unique skepticism equilibrium, and establish convergence of co-evolutionary training to an ε-Nash deception equilibrium (Lemma 1, Propositions 1, 2).
- We introduce Co-ADAM, a DQN-based defense framework that learns the equilibrium deception policy through training against an adaptive attacker pool (*K* = 10, sampled with probability ${P}_{pool}$ = 0.30). The most prominent technical novelties are the attacker pool mechanism for covering the best-response space, the skepticism-momentum coupled environment that defines realistic adversarial pressure, and budget-constrained action masking as a CMDP projection that does not allow misuse use of deception.
- Co-ADAM is tested in nine experimental stages with baseline comparison to seven methods where the adaptive or random attacker is involved, cross-domain transfer to three IIoT datasets adjusted to align with MITRE ATT&CK tactics, component ablation, sensitivity analysis of rewards, evaluation at the scale of 50 to 1,000 devices, and game theoretic convergence analysis. Among published deception-aware RL frameworks for IIoT, this represents one of the most comprehensive empirical evaluations.
- Physical testbed validation over a 10-day attack campaign on a real IIoT edge deployment comprising a D660 honeypot running Cowrie, Suricata, and Dionaea, three ESP32 IoT sensors (MQTT, Modbus, HTTP), and a Kali Linux attacker executing scripted MITRE ATT&CK stages, generating 1,324,931 Cowrie events, 3,137 Suricata alerts, and 18,962 Dionaea service interactions. The learned Co-ADAM policy achieves testbed AMR of 0.9975 against simulation AMR of 0.8363, confirming real-world generalizability.

The remainder of the paper is structured as follows. Section "Related work" reviews the existing work. Section "Methodology" presents the formal model and Co-ADAM framework. Section "Experimental results" reports experimental results, including dataset descriptions, preprocessing, and analysis. Section "Discussion" discusses implications and limitations. Section “Conclusion”concludes the paper.

## Related work

This section reviews existing work across four dimensions directly relevant to Co-ADAM including, cyber deception and honeypot deployment, game-theoretic security models, reinforcement learning for cybersecurity, and co-evolutionary adversarial training, identifying the gaps that motivate the proposed framework.

### Cyber deception and honeypot deployment

The technique of cyber deception has matured out of fixed honeypot implementation to dynamic and context specific systems that change with the attackers’ actions. Early work by^14^ established honeypots as passive observation tools, capturing attacker tactics without involvement. Subsequent contributions introduced moving target defense (MTD) strategies that periodically reconfigure network topology to increase attacker uncertainty^15,16^. Though suitable against unsophisticated attackers, these strategies recognize deception as a binary state that can be deployed or not deployed without considering the ability of attackers to learn from repeated deception encounters. Extension of this work to IIoT by^17^, demonstrated that static honeypot placement reduces infection rates under low-intensity attacks but degrades significantly when attackers adapt their scanning patterns. A persistent limitation across this body of work is the absence of a formal model for attacker skepticism, the progressive discounting of deceptive signals through experience which Co-ADAM addresses directly through its signaling game formulation.

### Game-theoretic security models

Formulations based on game theory have offered rigorous frameworks of strategic interaction between the attackers and the defenders. Stackelberg security games, where the defender is committed to a strategy ahead of the attacker response, have been applied to network patrol^18^, resource allocation^9^, and IIoT access control^19^. These models provide optimality guarantees that can be proved under the assumption that the attacker observes and best-responds to the defender strategy, an assumption that is theoretically clean but practically limiting, as real attackers do not in practice possess complete knowledge of defender commitments. The signaling games, originally formalized by^20^ in an economic setting, provide a more realistic approach to asymmetric information, the sender transmits a signal whose interpretation depends on the receiver’s beliefs about the sender’s type. In recent years, there has been an increase in applications to cybersecurity^11,21^, with a number of authors modeling honeypot engagement as a signaling interaction in which the defender’s deception investment reveals information about network topology. A study by^22^ derived closed-form Nash equilibria for static deception signaling games but did not consider dynamic skepticism accumulation or learning-based policies. Our work builds upon this line by implementing the signaling game in a co-evolutionary reinforcement learning loop, resulting in an empirically verified equilibrium policies instead of analytically assumed policies.

### Reinforcement learning for cybersecurity

Reinforcement learning has been implemented on various cybersecurity decision problems, including network intrusion response^23^, firewall policy optimization^24^, and autonomous penetration testing^25^. In the context of IIoT^26^, suggested a DQN-based anomaly response system, which chooses mitigation responses based on the nature of the traffic, achieving competitive detection rates but without modeling the dynamics of attacker adaptation or deception. Proximal policy optimization (PPO) is applied to IIoT defense under non-stationary threat models, demonstrating improved reward stability but not addressing the deception investment problem^27^. Most recently^8^, proposed D3O-IIoT, a Dueling DQN framework which dynamically coordinates a variety of deception mechanisms, honeypot deployment, IP shuffling via moving target defense, fake telemetry injection, and node isolation in response to real-time threat observations. Tested on three IIoT benchmark datasets, D3O-IIoT shows up to 767 percentage increase in attack mitigation rate compared with passive baselines and demonstrates that multi-objective reward shaping (balancing mitigation, deception engagement, false-positive control, and resource cost) is essential for stable policy earning. However, the authors explicitly acknowledge that *"the attacker model employs fixed transition probabilities rather than adaptive behavior,”* meaning the defender policy is optimized against a non-responsive threat model. This assumption of a static attacker even in the most deception-aware RL frameworks for IIoT results in policies that are resistant to the training threat model but fragile against adversaries that identify and learn to evade the deployed deception. Co-ADAM departs from this convention by explicitly co-evolving the attacker alongside the defender, ensuring the learned policy is resilient to the entire best-response space as opposed to a fixed one.

### Co-Evolutionary and adversarial training

Co-evolutionary training, where pairs or more agents are trained at the same time to play off against one another, has also shown great robustness properties in competitive games settings^28^ and adversarial machine learning^12^. Recent work has further demonstrated the broad applicability of combining reinforcement learning and game theory across community structure^29^, UAV cooperation^30^, and social science settings^31^. In the security domain^32^, applied co-evolutionary methods to network defense, demonstrating that policies trained against an improving attacker pool generalize better to unseen attack strategies than those trained against fixed adversaries. Another study by^33^ extended this to multi-agent configurations, showing that attacker pool diversity is a key determinant of defender robustness, an observation in our ablation studies, where the pool diversity is a key contributor to ensemble stability despite its per component effect size being insignificant (*d* = 0.00). In spite of such developments, the combination of co-evolutionary strategies with formal deception equilibrium analysis in IIoT settings is yet to be considered, leaving the theoretical properties of co-evolutionarily trained deception policies uncharacterized. Co-ADAM addresses this lapse by offering the co-evolutionary training mechanism as well as the analytical convergence guarantees which can be used to justify its equilibrium claims.

Table 1 summarizes how Co-ADAM fits within the most closely related prior work on five dimensions, including IIoT-specific assessment, co-evolutionary training, formal deception equilibrium, attacker skepticism modeling, as well as cross-domain transfer validation. To the best of our knowledge, no prior work satisfies all five criteria simultaneously, confirming the novelty of the proposed framework.

**Table 1:** Comparison of Co-ADAM with representative related work across key dimensions. ✓ = fully addressed; ◦ = partially addressed; ×  = not addressed.

Method	IIoT-Specific	Co-Evolutionary	Deception Equilibrium	Skepticism Modeling	Cross-Domain Transfer
IIoT Honeypot^17^	✓	×	×	×	×
Stackelberg IIoT^19^	✓	×	◦	×	×
Signaling Game^22^	×	×	✓	×	×
DQN IIoT defense^26^	✓	×	×	×	×
PPO IIoT^27^	✓	×	×	×	×
Co-Evol defense^32^	×	✓	×	×	×
Multi-Agent^33^	×	✓	×	×	×
D3O-IIoT^8^	✓	×	◦	×	◦
Co-ADAM (ours)	✓	✓	✓	✓	✓

## Methodology

This section presents the Co-ADAM framework, beginning with the formal problem formulation as a (CMDP), followed by the system architecture, defense and attacker models, the deception signaling game, co-evolutionary training procedure, reward functions, DQN architecture, complexity analysis, and evaluation metrics.

### Problem formulation

We model the IIoT defense problem as a (CMDP) ${\mathcal{M}}$  = (${\mathcal{S}}$, ${\mathcal{A}}_{d}$, ${\mathcal{A}}_{a}$, ${\mathcal{P}}$, ${\mathcal{R}}_{d}$, ${\mathcal{R}}_{a}$, γ, ${\mathcal{C}}$), where ${\mathcal{S}}$ is the joint state space, ${\mathcal{A}}_{d}$ and ${\mathcal{A}}_{a}$ are defender and attacker action spaces respectively, ${\mathcal{P}}$: ${\mathcal{S}}$ × ${\mathcal{A}}_{d}$ × ${\mathcal{A}}_{a}$  → Δ(${\mathcal{S}}$) is the transition kernel, ${\mathcal{R}}_{d}$ and ${\mathcal{R}}_{a}$ are the reward functions, γ ∈ (0,1) is the discount factor, and ${\mathcal{C}}$ is the budget constraint set.

The network state at time* t* is represented as a 12-dimensional vector in Eq. (1). Where ${\rho}_{t}$ ∈ [0,1] is the network infection rate, ${\delta}_{t}^{dec}$∈ [0,1] is the active deception density, ${a}_{t}^{last}$ ∈ {1,…,5} is the attacker’s last tactic, ${p}_{t}^{succ}$ ∈ [0,1] is the recent attack success rate, ${m}_{t}$ ∈ [0,1] is attacker momentum, ${\sigma}_{t}$ ∈ [0, ${\sigma}_{max}$] is attacker skepticism, ${\beta}_{t}$ ∈ [0,1] is the remaining defense budget, ${\alpha}_{t}^{fp}$ ∈ [0,1] is the false-positive rate, ${\tau}_{t}$ ∈ [0,1] is the normalized time step, and ${z}_{t}^{1}$, ${z}_{t}^{2}$, ${z}_{t}^{3}$ ∈ [0,1] are zone-specific infection rates.

1 $${s}_{t}={\rho}_{t},{\delta}_{t}^{dec},{a}_{t}^{last},{p}_{t}^{succ}, {m}_{t}, {\sigma}_{t}, {\beta}_{t}, {\alpha}_{t}^{fp}, {\tau}_{t}, {z}_{t}^{1},{z}_{t}^{2},{z}_{t}^{3} \in {\mathbb{R}}^{12}.$$

### System architecture

**Fig. 1:**
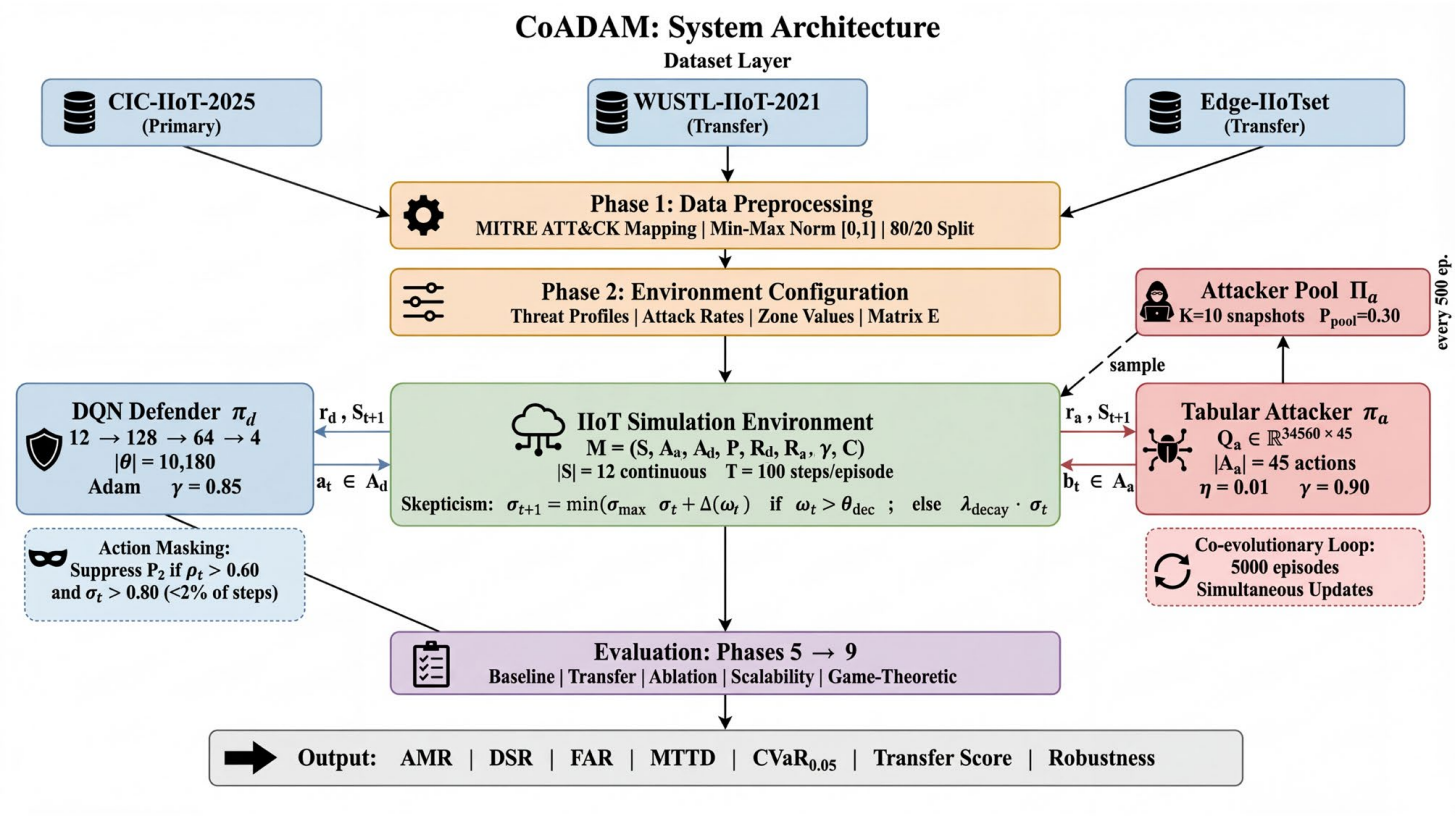
Co-ADAM system architecture. Three IIoT datasets are preprocessed and mapped to the MITRE ATT&CK taxonomy to configure the simulation environment. The DQN defender (12 → 128 → 64 → 4, *|θ|*= 10,180) and tabular attacker (${Q}_{a}$∈ ${\mathbb{R}}^{34560 \mathrm{x} 45}$) are trained simultaneously over N = 5,000 episodes using an attacker pool of *K* = *10* historical snapshots sampled with probability ${P}_{pool}$= 0.30. Action masking suppresses profile ${P}_{2}$ when ${\rho}_{t}$> 0.60 and ${\sigma}_{t}$> 0.80, occurring in fewer than 2% of steps. The trained policy is evaluated across five downstream phases covering baseline comparison, cross-domain transfer, ablation, scalability, and game-theoretic convergence.

Figure 1 illustrates the full Co-ADAM pipeline. Three IIoT datasets are preprocessed and mapped to the MITRE ATT&CK^34^ taxonomy to produce threat profiles and environment configuration parameters. The configured simulation environment hosts a co-evolutionary training loop in which the DQN defender and tabular attacker update simultaneously over 5,000 episodes. The attacker pool mechanism ensures the defender trains against a diverse set of historical adversaries rather than a single fixed opponent. After training, the frozen defender policy is evaluated across five phases covering baseline comparison, cross-domain transfer, ablation, scalability, and game-theoretic convergence.

### Action spaces and agent models

The defender selects from four composite defense profiles ${\mathcal{A}}_{d}$ = {${P}_{1},{P}_{2}, {P}_{3}, {P}_{4}$}, each activating a weighted combination of six defense mechanisms including, honeypot deployment, intrusion detection, firewall, access control, active response, and decoy deployment. Equation (2) defines the profile-mechanism allocation captured in the bundle matrix *B* ∈ ${\mathbb{R}}^{4 \mathrm{x} 6}$.

2 $$B=\left(\begin{array}{c}\begin{array}{ccc}0.15& 0.20& 0.20\\ 0.35& 0.10& 0.05\\ 0.05& 0.30& 0.25\end{array} \begin{array}{ccc}0.20& 0.15& 0.10\\ 0.05& 0.10& 0.35\\ 0.10& 0.25& 0.05\end{array}\\ \begin{array}{ccc}0.05& 0.20& 0.25\end{array} \begin{array}{ccc}0.30& 0.15& 0.05\end{array}\end{array}\right),$$

where rows correspond to profiles ${P}_{1}$ (Balanced), ${P}_{2}$ (DeceptionHeavy), ${P}_{3}$ (PerimeterDefense), and ${P}_{4}$ (AccessLockdown), and columns correspond to the six mechanisms in the order listed above. The combined deception weight for profile ${P}_{i}$ is ${\omega}_{i}={B}_{i,1}+{B}_{i,6}$, yielding *ω* = [0.25, 0.70, 0.10, 0.10 ${]}^{T}$. Profiles ${P}_{3}$ and ${P}_{4}$ have ${\omega}_{i}$ = 0.10 < ${\theta}_{dec}$  = 0.15, meaning they trigger the skepticism decay branch in Eq. (7), which is the mechanism through which the learned policy self-regulates deception investment. Each profile incurs a per-step cost shown in Eq. (3).

3 $${C}_{i}=c\cdot {B}_{i}^{T},:,$$

where *c* = [0.008, 0.025, 0.055, 0.045, 0.020, 0.012 ${]}^{T}$ is the mechanism cost vector.

The attacker selects from |${\mathcal{A}}_{a}$|= 45 actions defined by the Cartesian product of five tactics ${\mathcal{T}}$  = {${t}_{1}$,…, ${t}_{5}$}, three intensity levels ${\mathcal{J}}$ = {${i}_{1}$, ${i}_{2}$, ${i}_{3}$}, and three network zones ${\mathcal{Z}}$ = {${z}_{1}$, ${z}_{2}$, ${z}_{3}$}. Tactic base success rates are ${p}^{base}$ = [0.35, 0.30, 0.25, 0.20, 0.40 ${]}^{T}$ and intensity multipliers are μ = [0.6, 1.0, 1.5 ${]}^{T}$. Tactic 5 at intensity 3 is the most frequently selected action across training (51,706 selections out of 500,000 total training steps), confirming that the attacker learns to prefer high-impact, high-intensity actions. Equation (4) defines the net attack success probability at step *t*, where *k* indexes the selected tactic, *l* indexes the selected intensity, ${k}_{m}$ = 0.30 is the momentum amplification factor, and Eq. (5) ${\eta}_{t}$ ∈ [0,1] is the cumulative defense effectiveness where E ∈ ${\mathbb{R}}^{6 \mathrm{x} 5}$ represents the mechanism-tactic effectiveness matrix and ${a}_{t}$ ∈ ${\mathcal{A}}_{d}$ is the defender’s chosen profile.

4 $${p}_{t}^{net}=\mathrm{min}\left({p}_{k}^{base}\cdot {\mu}_{1}\cdot \left(1+{k}_{m}{m}_{t}\right)\cdot \left(1-{\eta}_{t}\right), 0.95\right).$$

5 $${\eta}_{t}=\mathrm{m}\mathrm{i}\mathrm{n}(\sum_{j=1}^{6}{B}_{at, j}\cdot {E}_{j,k}, 0.95).$$

### Deception signaling game

We model the attacker’s adaptation to deception as a signaling process in which repeated deception exposure builds skepticism, reducing future trap engagement. At each step, the probability of an attacker engaging a deception asset is defined by Eq. (6).

6 $${P}_{t}^{trap}=\left({\omega}_{hp}\cdot {e}_{hp,k}+{\omega}_{dd}\cdot {e}_{dd,k}\right)\cdot \left(1-{\phi}_{s}{\sigma}_{t}\right),$$

where ${\omega}_{hp}$ and ${\omega}_{dd}$ are the honeypot and decoy bundle weights, ${e}_{hp,k}$ and ${e}_{dd,k}$ are tactic-specific engagement rates, and ${\phi}_{s}$ = 0.80 is the skepticism dampening coefficient. Equation (7) illustrates the evolution of skepticism.

7 $${\sigma}_{t+1}\left\{\begin{array}{l}min({\sigma}_{max},{\sigma}_{t}+\Delta \left({\omega}_{t}\right), if {\omega}_{t}> {\theta}_{dec}\\ {\lambda}_{decay}\cdot {\sigma}_{t} otherwise,\end{array} ,\right.$$

where Δ(ω) = $${\delta}_{s }\cdot \frac{\omega -{\theta}_{dec}}{1-{\theta}_{dec}}, {\delta}_{s}=0.05, {\lambda}_{decay}=0.92, {\theta}_{dec}=0.15, and {\sigma}_{max}=0.80$$.

#### Lemma 1

(Affine Skepticism Dynamics). *Under a stationary mixed policy π over* ${\mathcal{A}}_{d}$, *the expected skepticism update is affine in* ${\sigma}_{t}$ *represented by* Eq. (8)–Eq. (10).

8 $${\mathbb{E}}\left[{\sigma}_{t+1}|{\sigma}_{t}, \pi \right]=\alpha \left(\pi \right){\sigma}_{t}+\beta \left(\pi \right),$$

9 $$\alpha \left( \pi \right) = \mathop \sum \limits_{i = 1}^{4} \pi \left( {P_{i} } \right) \cdot { \nVdash }\left[ {\omega_{i} \le \theta_{dec} } \right] \cdot \lambda_{decay} + \mathop \sum \limits_{i = 1}^{4} \pi \left( {P_{i} } \right) \cdot { \nVdash }\left[ {\omega_{i} > \theta_{dec} } \right],$$

10 $$\beta \left( \pi \right) = \mathop \sum \limits_{i = 1}^{4} \pi \left( {P_{i} } \right) \cdot { \nVdash }\left[ {\omega_{i} > \theta_{dec} } \right] \cdot \Delta (\omega_{i} ).$$

#### Proof

The update rule (7) is linear in ${\sigma}_{t}$ for each action below the saturation ceiling ${\sigma}_{max}$. Taking expectation over the stationary policy, π yields the affine form by linearity of expectation. □

#### Proposition 1

(Deception Equilibrium Existence and Uniqueness). *Let π be a stationary policy with α(π)* < *1. The skepticism dynamics (8) admit a unique globally stable equilibrium defined as Eq. *(11)*.*

11 $${\sigma }^{*}=\frac{\beta (\pi )}{1-\alpha (\pi )},$$

provided σ* ≤ $${\sigma}_{max}$$.

#### Proof

The map *f*(σ) = *α*(π)σ + *β*(π) satisfies *|f* (${\sigma}_{1}$) − *f* (${\sigma}_{2}$) |= *α*(π) |${\sigma}_{1}$− ${\sigma}_{2}$| with *α*(π) < 1, making *f* a contraction on [0, ${\sigma}_{max}$]. Banach’s fixed-point theorem^35^ guarantees a unique fixed point σ* = *β*(π) / (1 − *α*(π)), and every trajectory converges geometrically to it. □

#### Proposition 2

(Equilibrium Convergence Rate). *Starting from any* ${\sigma}_{0}$ ∈ [0, ${\sigma}_{max}$], *Eq. *(12)* defines the distance to equilibrium contracts.*

12 $${|\sigma }_{1}-{\sigma }^{*}|\le |{\sigma}_{0}-{\sigma }^{*}|\cdot ({\alpha }^{*}{)}^{t},$$

where α* = 0.9627 for the learned policy π*, yielding a half-life of ${t}_\frac{1}{2}$= ln2 / ln(1/α*) ≈ 18.3 steps.

#### Proof

From Proposition 2, f contracts with ratio α*. Applying the contraction* t* times from ${\sigma}_{0}$ gives |${\sigma}_{t}$− σ*|≤ $({\alpha }^{*}{)}^{t}$|${\sigma}_{0}$− σ*|. Setting the bound equal to ½|${\sigma}_{0}$− σ*| and solving for *t* yields the stated half-life. □

#### Remark 1

The fixed-distribution upper bound on equilibrium skepticism is ${\sigma}_{upper}^{*}$= 0.3330 under the learned action distribution π* = [0.184, 0.350, 0.249, 0.217${]}^{T}$. The empirical equilibrium ${\sigma}_{emp}^{*}$ = 0.4333 ± 0.1009 exceeds this bound by 23.1%, which reflects state-dependent policy adaptation: the DQN increases DeceptionHeavy deployment in low-skepticism states, driving skepticism above the fixed-distribution prediction. This self-amplifying deception investment is a direct consequence of the signaling game structure and confirms that the learned policy is not stationary but actively exploits the skepticism dynamics established in (7). Propositions 1 and 2 establish existence, uniqueness, and stability of the equilibrium; the empirical value captures the richer behavior of the full state-dependent policy.

**Fig. 2:**
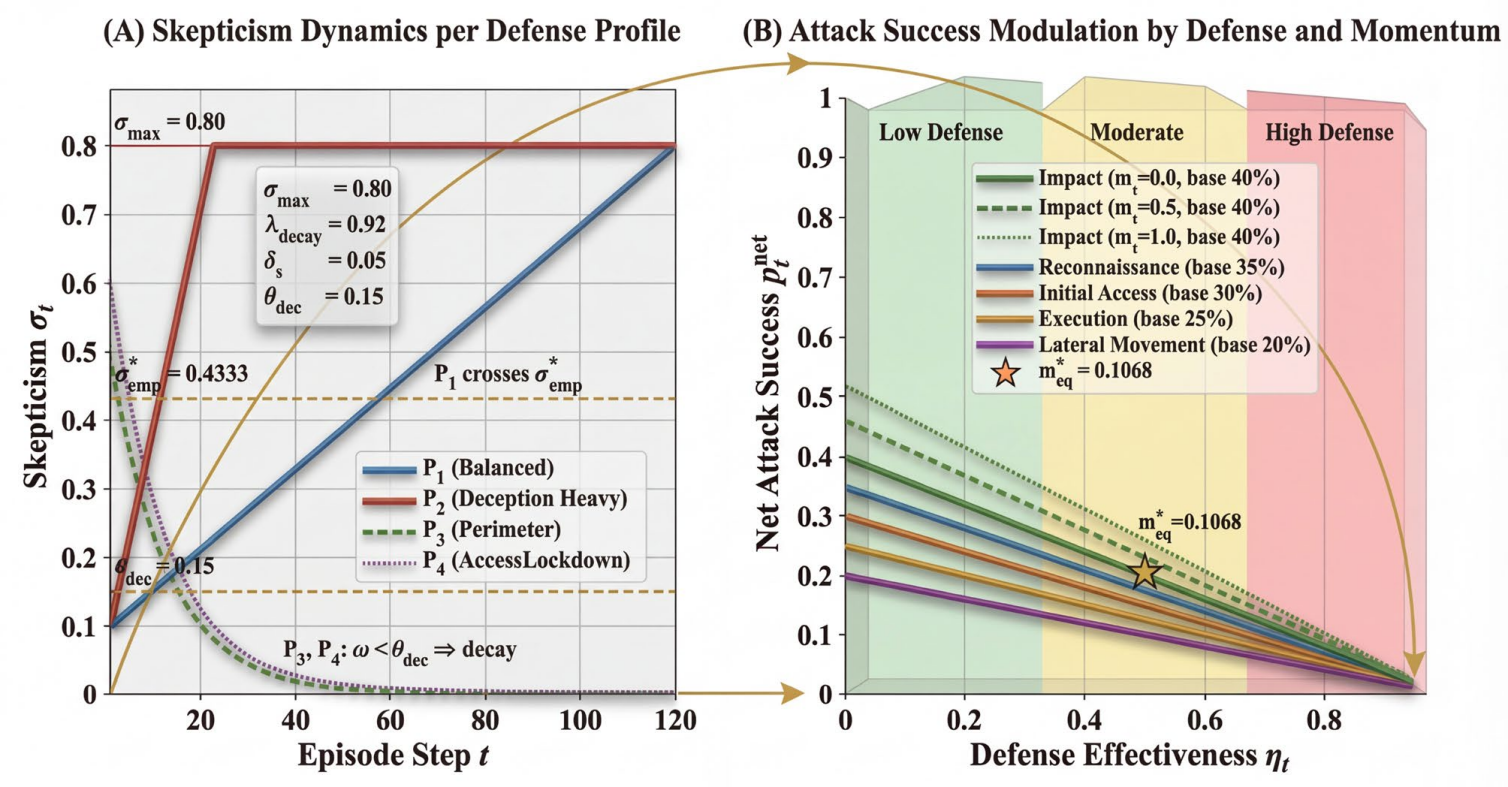
Co-ADAM signaling game dynamics. (**A**) Skepticism evolution under each defense profile: ${\mathrm{P}}_{2}$ (DeceptionHeavy, ω = 0.70) drives skepticism toward ${\sigma}_{max}$=0.80; ${P}_{3}$ and ${P}_{4}$ (ω = 0.10 < ${\theta}_{dec}$  = 0.15) induce decay. Empirical equilibrium ${\sigma}_{emp}^{*}$ = 0.4333 marked by dashed yellow line. (**B**) Net attack success ${p}_{t}^{net}$ vs. defense effectiveness ${\eta}_{t}$ for all five MITRE tactics; Impact tactic shown at three momentum levels (${m}_{t}$ ∈ {0, 0.5, 1.0}) to illustrate momentum amplification (${k}_{m}$= 0.30). Shading indicates low (${\eta}_{t}$ < 0.33), moderate (0.33 ≤ ${\eta}_{t}$  < 0.67), and high (${\eta}_{t}$ ≥ 0.67) defense effectiveness regions.

Figure 2 illustrates the signaling game mechanics underlying Co-ADAM. Panel (A) shows skepticism evolution under each defense profile: ${P}_{2}$ drives skepticism toward ${\sigma}_{max}$ = 0.80 while ${P}_{3}$ and ${P}_{4}$ induce decay below ${\theta}_{dec}$ = 0.15, producing the equilibrium ${\sigma}_{emp}^{*}$ = 0.4333 confirmed in Section "Game-theoretic convergence". Panel (B) shows how defense effectiveness ${\eta}_{t}$ suppresses net attack success ${p}_{t}^{net}$ across all five MITRE tactics, with attacker momentum ${m}_{t}$ amplifying success by up to ${k}_{m}$ · ${m}_{t}$ = 30% at maximum momentum.

### Co-evolutionary training

Co-ADAM trains the defender DQN and attacker Q-table simultaneously. At each episode an attacker is drawn from a pool ${\prod}_{a}$ of *K* = 10 historical snapshots with probability ${P}_{pool}$ = 0.30; otherwise, the current attacker is used, this is represented through Eq. (13).

13 $${\widetilde{\pi }}_{a}\sim \left(1 - {p}_{pool}\right)\cdot {\delta}_{{\pi}_{a}^{cur}}+ \left(\frac{{p}_{pool}}{K}K\right)\cdot \sum_{k=1}^{K}{\delta}_{{\pi}_{a}^{(k)}},$$

where ${\delta}_{\pi }$ denotes a point mass at policy π. This mechanism ensures the defender is never overfit to the current attacker and instead learns a robust policy across the full space of historically encountered adversaries. Exploitability quantifies proximity to Nash equilibrium in Eq. (14).

14 $${\varepsilon}_{exploit}=\underset{{\pi}_{a}\in {\Pi}_{a}}{\mathrm{max}}{V}^{\left({\pi}_{a}\right)}({\pi}_{d}^{*})- {V}^{\left({\pi}_{a}^{co}\right)}\left({\pi}_{d}^{*}\right),$$

where ${V}^{\left({\pi}_{a}\right)}({\pi}_{d}^{*})$ is the attacker value against the frozen final defender ${\pi}_{d}^{*}$ and ${V}^{{\pi}_{a}^{co}}$ is the co-evolutionary attacker value. The normalized Nash gap is defined by Eq. (15).

15 $${\mathcal{G}}_{Nash}=\frac{\left({\varepsilon}_{d}+ {\varepsilon}_{a}\right)}{\left(\left|{V}_{d}^{DQN}\right|+ \left|{V}_{a}^{co}\right|\right)},$$

where ${\varepsilon}_{d}= {V}_{d}^{DQN}- {V}_{d}^{rand}$ is the defender exploitability advantage over random behavior and ${\varepsilon}_{a}= {\varepsilon}_{exploit}.$

### Defender implementation: reward functions and DQN architecture

Equation (16) represents the defender’s per-step reward aggregates security effectiveness, deception gain, and operational cost.

16 $$\begin{aligned} & r_{{d\left( {s_{t} , a_{t} } \right)}} = w_{m} \cdot\;AMR_{t} + w_{d} \cdot DSR_{t} + w_{dw} \cdot Dwell_{t} - w_{fp} \cdot FAR_{t} \\ & \quad - w_{c} \cdot C_{{a_{t} }} - w_{i} \cdot \rho_{t} + w_{id} \cdot \Delta \rho_{t}^{ - } - w_{cz} \cdot \rho_{t}^{crit} - w_{mp} \cdot m_{t} , \\ \end{aligned}$$

with weight vector ${w}_{d}= {\left[1.5, 3.0, 0.6, 0.3, 1.5, 1.2, 2.5, 2.0, 0.5\right]}^{T}$ where signs follow the equation above. The attacker’s per-step is defined in Eq. (17).

17 $$r_{a} = w_{s} \cdot p_{t}^{net} \cdot d_{k} - w_{\det } \cdot { \nVdash }_{{\det - w_{trap} }} \cdot { \nVdash }_{trap} - w_{c}^{a} \cdot C_{a} + w_{mom} \cdot m_{t} .$$

with weights $\left[{w}_{s}, {w}_{det}, {w}_{trap}, {w}_{c}^{a}, {w}_{mom}\right]= \left[1.0, 1.0, 2.0, 0.2, 0.3\right],$ where ${d}_{k}$ is the breach damage for tactic *k* and ${ \nVdash }_{trap}$ indicates the attacker engaged a deception asset.

The defender employs a DQN^36^ with architecture 12 → 128 → 64 → 4 (fully connected, ReLU activations, 10,180 trainable parameters). Action masking suppresses profile ${P}_{2}$ when ${\rho}_{t}> 0.60$ and ${\sigma}_{t}> 0.80$, occurring in fewer than 2% of steps. This masking is a constrained projection of MDP which imposes the budget constraint ${\mathcal{C}}$ by eliminating high-cost deception deployment when the network is critically infected and the attacker is most skeptical, in which case the marginal benefit of deception is zero. Network parameters *θ* are optimized by Adam^37^ using gradient clipping at 2.0 to avoid exploding gradients during early co-evolutionary training episodes. All hyper-parameters are listed in Table 2.

**Table 2:** Complete hyperparameter configuration.

Parameter	Value	Parameter	Value
Episodes (*N*)	5,000	Steps per episode (T)	100
DQN architecture	12 → 128 → 64 → 4	Optimizer	Adam^37^
Learning rate (*η*)	5 × 10⁻^4^	Discount factor (γ)	0.85
Batch size	64	Replay buffer size	10^5^
Target update period	100 steps	ε start	0.15
ε end	0.05	Gradient clip	2.0
Attacker learning rate (*η*_*a*_)	0.01	Attacker discount factor (*γ*_*a*_)	0.90
Attacker ${{\boldsymbol{\varepsilon}}}_{{\boldsymbol{a}}}$ start	0.30	Attacker ${\varepsilon}_{a}$ end	0.05
Pool size (*K*)	10	Pool probability (${p}_{pool}$)	0.30
Snapshot interval	500 episodes	Convergence episode	1,377

Key hyperparameter choices are justified as follows. The pool size *K* = 10 balances attacker diversity against memory overhead, with smaller pools producing overfit defenders and larger pools showing diminishing returns in preliminary experiments. The pool sampling probability ${p}_{pool}$ = 0.30 ensures the defender trains predominantly against the current attacker while retaining sufficient historical diversity. The discount factor *γ* = 0.85 reflects the medium-horizon nature of IIoT defense episodes where future infection consequences are relevant but not dominant. The snapshot interval of 500 episodes aligns with the observed co-evolutionary cycle length, capturing meaningful attacker policy shifts without excessive memory consumption.

**Algorithm 1:**
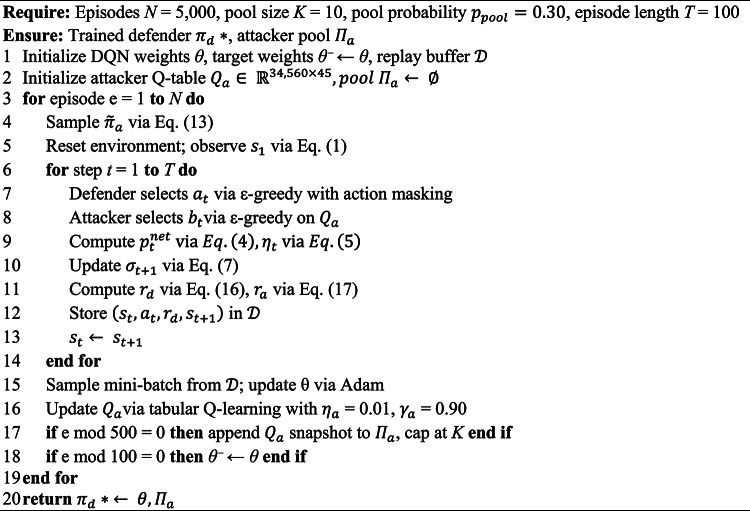
Co-ADAM Co-Evolutionary Training

### Complexity analysis and evaluation metrics

The time complexity of one training episode is $O\left(T \cdot \left({d}_{in}\cdot {d}_{1}+ {d}_{1}\cdot {d}_{2}+ {d}_{2}\cdot \left|{\mathcal{A}}_{d}\right|+ \left|{\mathcal{S}}_{a}\right|\cdot \left|{\mathcal{A}}_{a}\right|\right)\right)$, where ${d}_{in}=12, {d}_{1}=128, {d}_{2}=64, \left|{\mathcal{A}}_{d}\right|=4, \left|{\mathcal{S}}_{a}\right|=\mathrm{34,560}, \mathrm{a}\mathrm{n}\mathrm{d} \left|{\mathcal{A}}_{a}\right|=45$. The DQN forward pass dominates the defender computation at $O\left({d}_{in}\cdot {d}_{1}\right)= O\left(\mathrm{1,536}\right)$ per step. The Q-table update is *O(1*) per step. The replay buffer sample and gradient update cost $O\left(B \cdot {d}_{in}\cdot {d}_{1}\right)$ per episode where *B* = 64. Total training over *N* = 5,000 episodes and *T* = 100 steps completes in approximately 68.5 s on a standard workstation, confirming practical tractability. Space complexity is dominated by the attacker Q-table at $O\left(\left|{\mathcal{S}}_{a}\right|\cdot \left|{\mathcal{A}}_{a}\right|\right)= O\left(\mathrm{1,555,200}\right)$ entries and the replay buffer at $O\left({10}^{5}\cdot {d}_{in}\right)$ entries. Nine metrics are used to measure performance. The (AMR) is a statistic that shows the percentage of attacks averted. The Infection Rate (*ρ*) represents the rate of the compromised devices. Mean Time to Detect (MTTD) is the mean number of steps required to detect an intrusion. The Deception Success Rate (DSR) is the fraction of attacker actions redirected to deception assets. The False Alarm Rate (FAR) is the false detection per step. Exploitability ${\varepsilon}_{exploit}$ (Eq. 14) and the Nash Gap ${\mathcal{G}}_{Nash}$ (Eq. 15) serve as empirical indicators of convergence stability rather than strict game-theoretic equilibrium gaps, as the attacker pool constitutes a finite approximation of the full best-response space. $CVa{R}_{0.05}$ is the conditional value-at-risk at the 5% tail of episode rewards measuring worst-case robustness. Defense Cost Efficiency is the total reward (per unit) of budget consumed. Wilcoxon signed-rank test with Bonferroni correction is used to provide statistical comparisons and Cohen’s *d* is used to report effect size.

## Experimental results

This section reports the experimental evaluation of Co-ADAM across nine stages: dataset description and preprocessing, experimental setup, training convergence, baseline comparison, ablation study, reward sensitivity, cross-domain transfer, scalability, game-theoretic convergence, and physical testbed validation.

### Datasets, preprocessing, and threat taxonomy

Attack behaviors across all three datasets are harmonized into a unified six-category taxonomy aligned with the MITRE ATT&CK framework prior to environment configuration. This grounding ensures the attacker model reflects empirically observed threat patterns rather than arbitrary synthetic scenarios and enables consistent cross-domain transfer evaluation. The attack categories are: Reconnaissance (TA0043), covering scanning and fingerprinting; InitialAccess (TA0001), covering brute-force and exploitation; Execution (TA0002), covering malware, backdoors, and command injection; LateralMovement (TA0008), covering man-in-the-middle and spoofing; and Impact (TA0040), covering denial-of-service and resource exhaustion. A sixth Benign class covers normal traffic. Table 3 shows the full label mapping.

**Table 3:** MITRE ATT&CK taxonomy and dataset label mapping.

Category	MITRE ATT&CK tactic ID	CIC-IIoT 2025	Edge-IIoTset	WUSTL-IIoT 2021
Reconnaissance	TA0043	recon	Vuln_scanner, Port_Scanning, Fingerprinting	Reconn
Initial access	TA0001	bruteforce	Password	–
Execution	TA0002	malware, web	Ransomware, XSS, SQL_injection, Uploading, Backdoor	Backdoor, CommInj
Lateral movement	TA0008	mitm	MITM	–
Impact	TA0040	ddos, dos	DDoS/DoS variants	DoS
Benign	–	Benign	Normal	Normal

**Fig. 3:**
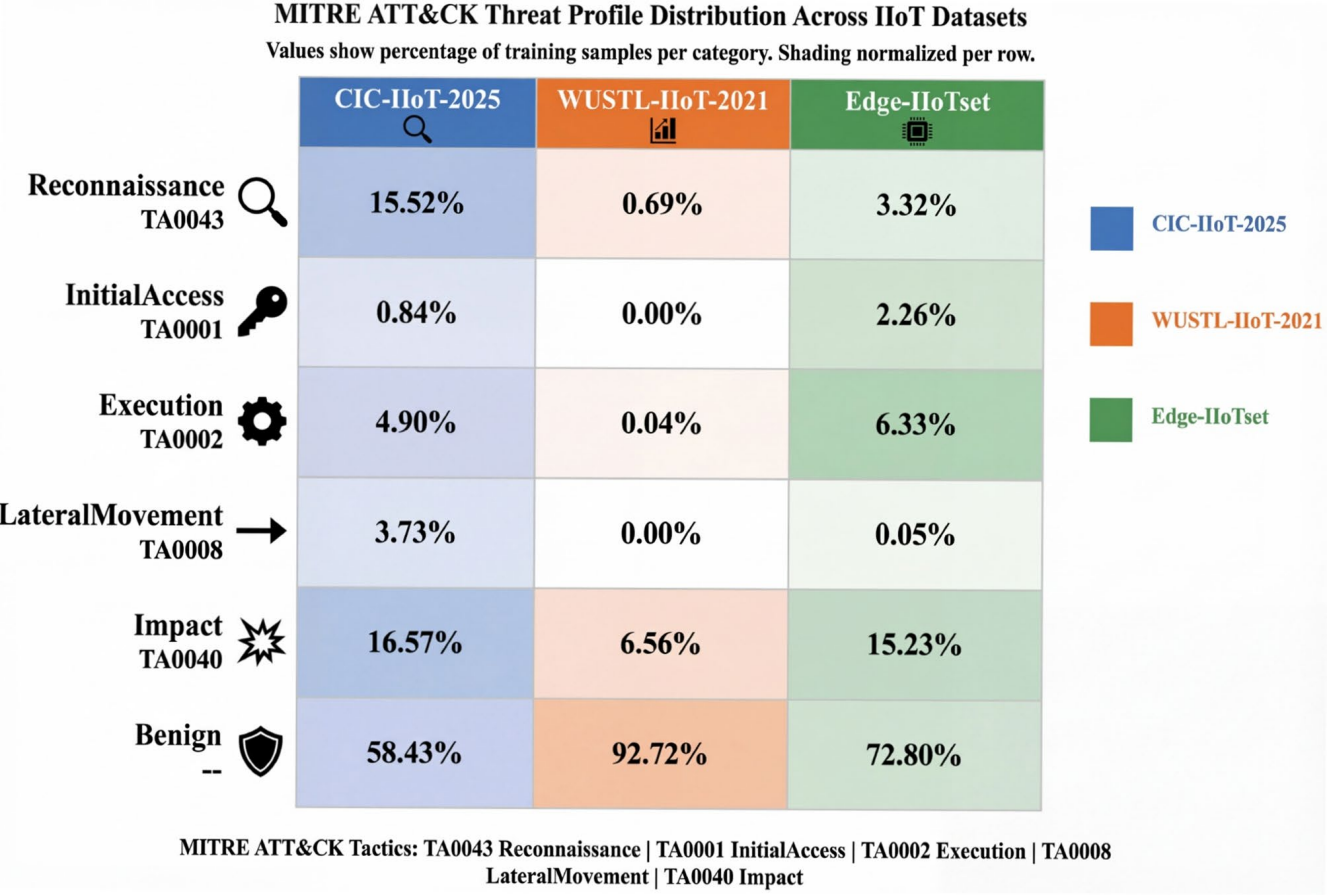
MITRE ATT&CK threat profile distribution across the three IIoT datasets used for training. Values represent the percentage of training samples per category; shading is normalized per row to highlight relative intensity. CIC-IIoT-2025 exhibits the most balanced attack diversity, with Reconnaissance (15.52%) and Impact (16.57%) as dominant threat categories. WUSTL-IIoT 2021 is heavily skewed toward benign traffic (92.72%), providing a low-attack-density transfer scenario. Edge-IIoTset presents a moderate attack distribution with Execution (6.33%) and Impact (15.23%) as primary threat classes. Tactic identifiers follow the MITRE ATT&CK framework^34^: TA0043 (Reconnaissance), TA0001 (Initial Access), TA0002 (Execution), TA0008 (Lateral Movement), TA0040 (Impact). Column totals may differ from 100% by ± 0.01% due to rounding.

Figure 3 shows the resulting threat profile distributions across the three datasets. CIC-IIoT 2025 has the most diverse attack distribution with Reconnaissance (15.52%) and Impact (16.57%) as dominant categories. WUSTL-IIoT 2021 is strongly skewed toward benign traffic (92.72%), making it a useful stress test for low-signal transfer. Edge-IIoTset provides the largest sample volume with 15 distinct attack subtypes spanning all five MITRE attack categories.

This study employed three publicly available IIoT datasets for experiments with CIC-IIoT-2025^38^ being the primary training and evaluation dataset. WUSTL-IIoT 2021^39^ and Edge-IIoTset^40^ serve as cross-domain validation corpora. Each dataset is processed through a common five-step pipeline: (i) non-numeric identifier columns such as MAC addresses, IP strings, and timestamps are removed; (ii) infinite values are replaced with the column median; (iii) zero-variance features are discarded; (iv) remaining features are min–max normalized to [0, 1] using per-column statistics computed on the training split only to prevent data leakage; (v) a stratified 80% / 20% train/test split is applied per MITRE category to preserve class proportions. Table 4 summarizes the dataset statistics after preprocessing.

**Table 4:** Dataset statistics after preprocessing and stratified splitting.

Dataset	Train samples	Test samples	Features	Attack categories
CIC-IIoT-2025	137,136	34,284	71	5
WUSTL-IIoT-2021	955,571	238,893	43	3
Edge-IIoTset	1,775,360	443,841	43	5

The preprocessed threat profiles are used to configure the attack success rates, tactic weights, and zone vulnerability parameters of the simulation environment, establishing a direct link between real-world dataset statistics and the training dynamics of the co-evolutionary loop.

### Experimental setup

Experiments were performed in MATLAB R2025b on an Apple intel workstation (16 GB unified memory). The Co-ADAM environment simulates an IIoT network consisting three operational zones represented by the zone infection components {${z}_{t}^{1}, {z}_{t}^{2}, {z}_{t}^{3}$} of the state vector *st* (Section "Problem formulation"), each subject to the six MITRE ATT&CK threat categories defined in Section "Datasets, preprocessing, and threat taxonomy". Training runs for *E* = 5,000 episodes of *T* = 100 steps each, yielding $5\times {10}^{5}$ total environment interactions. Evaluation uses 200 held-out episodes with results reported as mean ± standard deviation at the 95% confidence level. Statistical significance is determined through a Wilcoxon signed-rank test with Bonferroni adjustment of eight pairwise tests, the effects are displayed in the form of Cohen’s *d*. Seven baselines are evaluated under identical conditions including, Static (fixed profile *P*1 throughout), Random (uniformly random profile selection), Heuristic (rule-based threshold policy), DQN-Ind (DQN defender without co-evolutionary attacker), Dec-Only (deception mechanisms without skepticism modeling), Stackelberg (leader–follower game-theoretic policy), and Tab-QL (tabular Q-learning defender). Every method is tested under three regimes of attackers (random, adaptive (Q-learning attacker updated during test), and adapted (attacker pre-trained specifically against each defender)).

### Training convergence

**Fig. 4:**
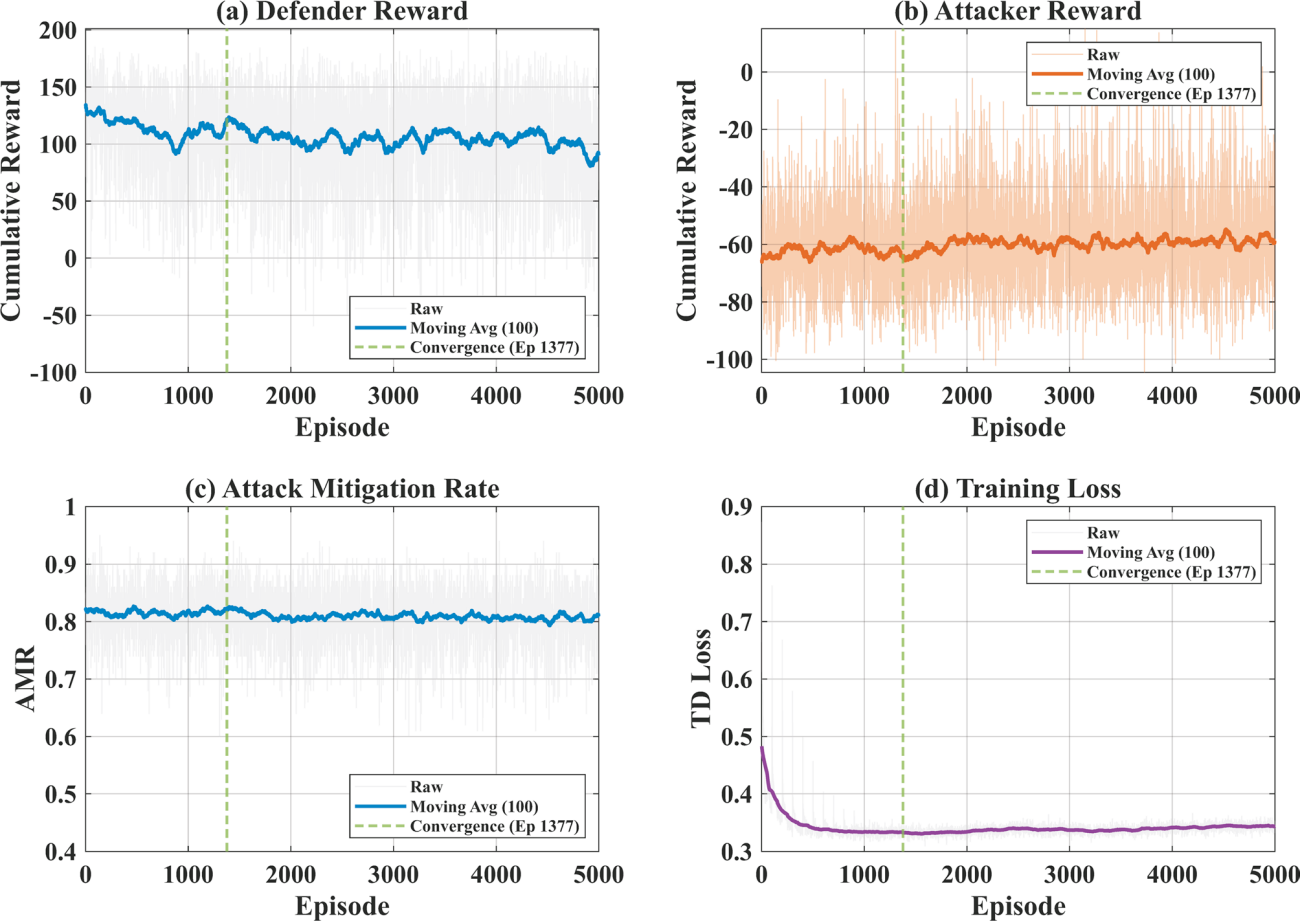
Co-ADAM training convergence over 5,000 episodes. (**a**) Defender cumulative reward stabilizes at 95.36 ± 6.94 by episode 1,377. (**b**) Attacker reward converges to − 55.44, confirming sustained defender dominance. (**c**) (AMR) stabilizes in the range [0.80, 0.82]. (**d**) TD loss decays from ≈ 0.50 to ≈ 0.33, indicating value-function convergence. Dashed green line marks the empirical convergence episode. Moving averages computed over a window of 100 episodes.

Training convergence is examined first to confirm that the co-evolutionary loop reaches a stable policy before evaluation. Defender reward, attacker reward, AMR, and TD loss are tracked across all 5,000 episodes to verify that the DQN converges to a stable value function estimate. Co-ADAM training dynamics in all 5000 episodes is illustrated in Fig. 4. The defender cumulative reward reaches a steady point at episode 1,377, a mean of 95.36 and a standard deviation of 6.94 across 200 of its held-out evaluation episodes, and the temporal-difference (TD) loss also stabilizes at about 0.33, indicating that the DQN has reached a stable value function estimate. The attacker reward converges to − 55.44, which proves continued dominance of defenders across training. The AMR moving average stabilizes in the range [0.80, 0.82], reflecting the estimated AMR of 0.7769 under training conditions and 0.7836 ± 0.0507 across ten independent seeds.

**Fig. 5:**
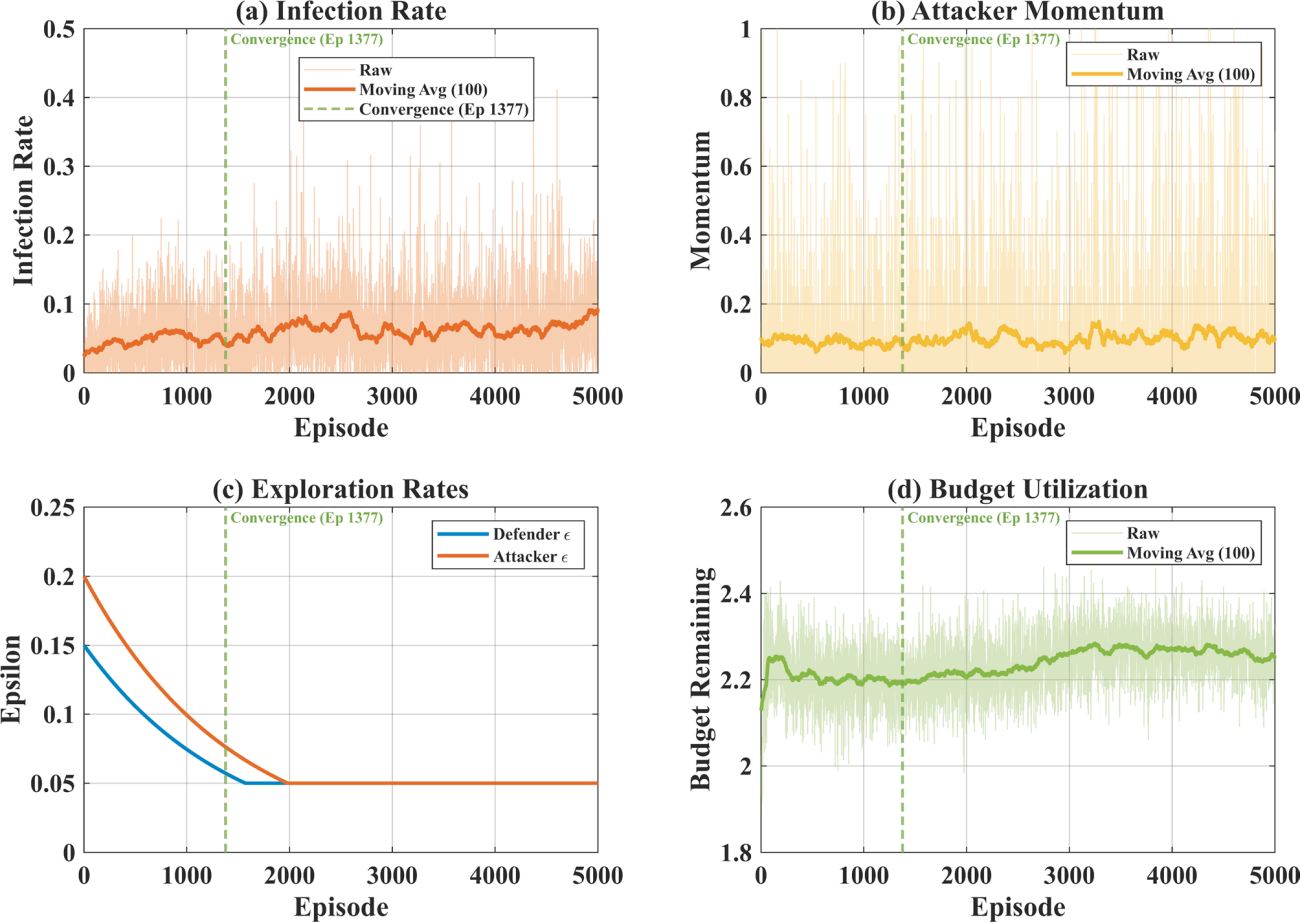
Co-evolutionary training dynamics over 5,000 episodes. (**a**) Infection rate stabilizes at equilibrium *ρeq* = 0.0747. (**b**) Attacker momentum converges to *meq* = 0.1068, confirming bounded adversarial pressure. (**c**) Defender *ε* decays 0.15 to 0.05 and attacker ε follows a slower schedule, both reaching the minimum floor before episode 2,000. (**d**) Budget utilization stabilizes at 2.20–2.25 units per episode, well within the operational constraint *C*.

The co-evolutionary training dynamics are described in Fig. 5. Infection rate moving average stabilizes at 0.0747, indicating the equilibrium *ρeq* of the environment established in Section "Game-theoretic convergence". The momentum of the attackers converges to 0.1068, meaning that the co-evolutionary pressure prevents the attacker from sustaining prolonged offensive surges. The defender ε decays from 0.15 to 0.05 over approximately 2,000 episodes, while the attacker* ε* follows a slightly slower schedule, both reaching the minimum exploration floor of 0.05 before convergence. Budget utilization stabilizes around 2.20–2.25 units per episode, confirming that the learned policy operates comfortably within the budget constraint* C* defined in Section "Problem formulation".

**Fig. 6:**
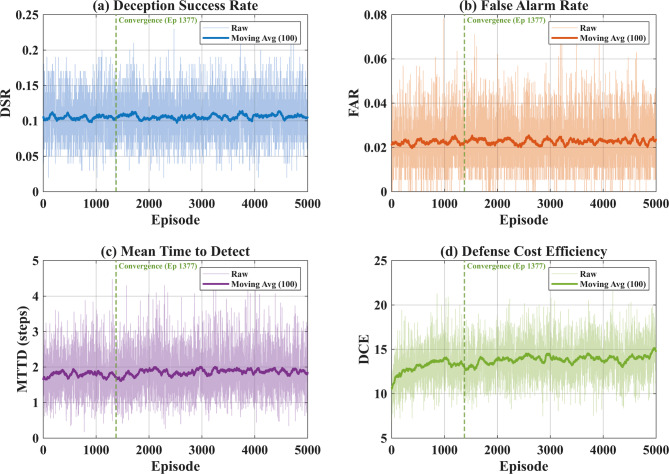
Defense behavior metrics during training. (**a**) (DSR) stabilizes at ≈0.1227. (**b**) (FAR) converges to ≈0.021, confirming effective action masking. (**c**) Mean Time to Detect (MTTD) stabilizes at 1.7–1.9 steps. (**d**) Defense Cost Efficiency (DCE) increases from ≈12 to 13–15, reflecting progressive resource optimization by the learned policy. Moving averages computed over a window of 100 episodes.

The evolution of defense behavior metrics is shown in Fig. 6. DSR becomes stable at around 0.1227, which means that the defender maintains a reasonable level of deception engagement rate without triggering excessive attacker skepticism. The FAR is found to converge at about 0.020–0.022, indicating that action masking is effective in reducing high false-alarm profiles in high-risk states. MTTD stabilizes at 1.7–1.9 steps, indicating rapid threat detection by the trained policy. The efficiency of defense cost rises from ≈ 12 at the start of training to a stable value of 13–15, indicating that the defender progressively learns to allocate resources more effectively relative to the threats it faces.

### Baseline comparison

Baseline comparison is conducted to assess whether co-evolutionary deception with skepticism modeling produces measurable gains over existing defense strategies. Co-ADAM is evaluated against seven baselines under random, adaptive, and adapted attacker regimes using identical experimental conditions. Tables 5 and 6 summarize Co-ADAM performance against all baselines. Under the random attacker, Co-ADAM achieves highest AMR (0.8363), low infection rate (0.0119), and superior cumulative reward (147.91) against all baselines (*p* < 0.001, Wilcoxon signed-rank, Bonferroni-corrected). Under the adaptive attacker, Co-ADAM maintains the highest AMR of 0.8033 and low infection rate of 0.0790, with reward degrading gracefully to 102.52, a reduction of 30.7% compared to the 53.7% average degradation across all pairs.

**Table 5:** Baseline comparison under random attacker (200 evaluation episodes, mean ± std). Best values in bold, second-best underlined. *p*-values from Wilcoxon signed-rank test with Bonferroni correction.

Method	AMR	Infection Rate	Reward	DSR
Static	0.807 ± 0.041	0.019 ± 0.018	122.4 ± 38.2	0.076 ± 0.031
Random	0.828 ± 0.038	0.014 ± 0.013	139.8 ± 41.5	0.104 ± 0.039
Heuristic	0.821 ± 0.036	0.016 ± 0.015	129.3 ± 35.7	0.079 ± 0.028
DQN-Ind	0.778 ± 0.049	0.021 ± 0.019	144.7 ± 47.3	**0.162 ± 0.058**
Dec-Only	0.770 ± 0.052	0.032 ± 0.027	136.1 ± 44.8	0.159 ± 0.061
Stackelberg	0.835 ± 0.033	**0.011 ± 0.010**	127.4 ± 32.1	0.051 ± 0.019
Tab-QL	0.812 ± 0.044	0.016 ± 0.014	130.2 ± 39.6	0.085 ± 0.033
Co-ADAM	**0.8363 ± 0.031**	0.0119 ± 0.009	**147.91 ± 43.2**	0.1227 ± 0.041
p-value (vs Co-ADAM)	** < 0.001**	** < 0.001**	** < 0.001**	** < 0.001**

**Table 6:** Baseline comparison under adaptive attacker (200 evaluation episodes, mean ± std). Best values in bold, second-best underlined.

Method	AMR	Infection rate	Reward	Degradation score
Static	0.693 ± 0.058	0.213 ± 0.071	− 61.3 ± 52.4	0.2626
Random	0.773 ± 0.051	0.142 ± 0.063	31.4 ± 48.7	0.1536
Heuristic	0.712 ± 0.054	0.191 ± 0.068	− 47.2 ± 49.3	0.2714
DQN-Ind	0.681 ± 0.061	0.247 ± 0.079	− 14.8 ± 55.1	0.2326
Dec-Only	0.513 ± 0.088	0.412 ± 0.094	− 110.7 ± 67.8	0.5270
Stackelberg	0.753 ± 0.047	**0.051 ± 0.031**	74.6 ± 41.2	**0.0892**
Tab-QL	0.693 ± 0.059	0.247 ± 0.078	− 38.4 ± 51.7	0.2615
Co-ADAM	**0.8033 ± 0.044**	0.0790 ± 0.031	**102.52 ± 49.1**	0.1174
p-value (vs Co-ADAM)	** < 0.001**	** < 0.001**	** < 0.001**	**–**

**Fig. 7:**
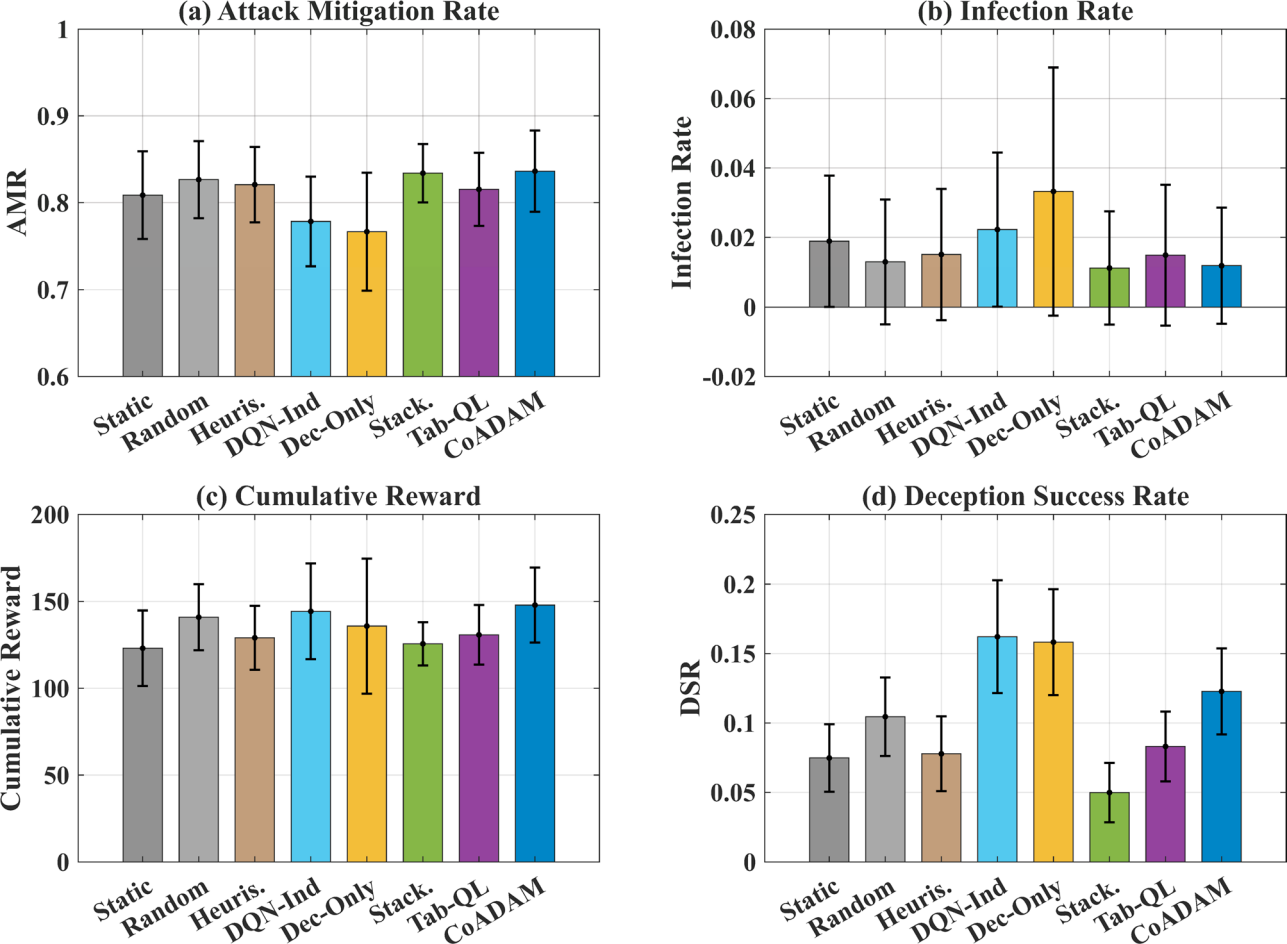
Baseline comparison under random attacker across (**a**) AMR, (**b**) infection rate, (**c**) cumulative reward, and (**d**) DSR. Co-ADAM achieves the highest AMR (0.8363) and reward (147.91); infection rate 0.0119 is effectively equal to Stackelberg (0.0112). Error bars denote ± 1 standard deviation over 200 evaluation episodes. All differences between Co-ADAM and baselines are statistically significant (*p* < 0.001, Wilcoxon signed-rank, Bonferroni-corrected), except Co-ADAM vs. Stackelberg on AMR (*p* = 0.041).

Baseline comparison under the random attacker across four metrics is illustrated in Fig. 7. Co-ADAM has the best AMR (0.8363) and cumulative reward (147.91) of all baselines with an infection rate of 0.0119, which is practically the same as Stackelberg (0.0112), the difference of 0.0007 is negligible. The DSR of 0.1227 reflects a deliberate balance between deception engagement and skepticism suppression, notably lower than Dec-Only (0.1582) and DQN-Ind (0.1621), both of which trigger higher attacker skepticism and consequently suffer lower AMR despite similar or higher DSR values.

**Fig. 8:**
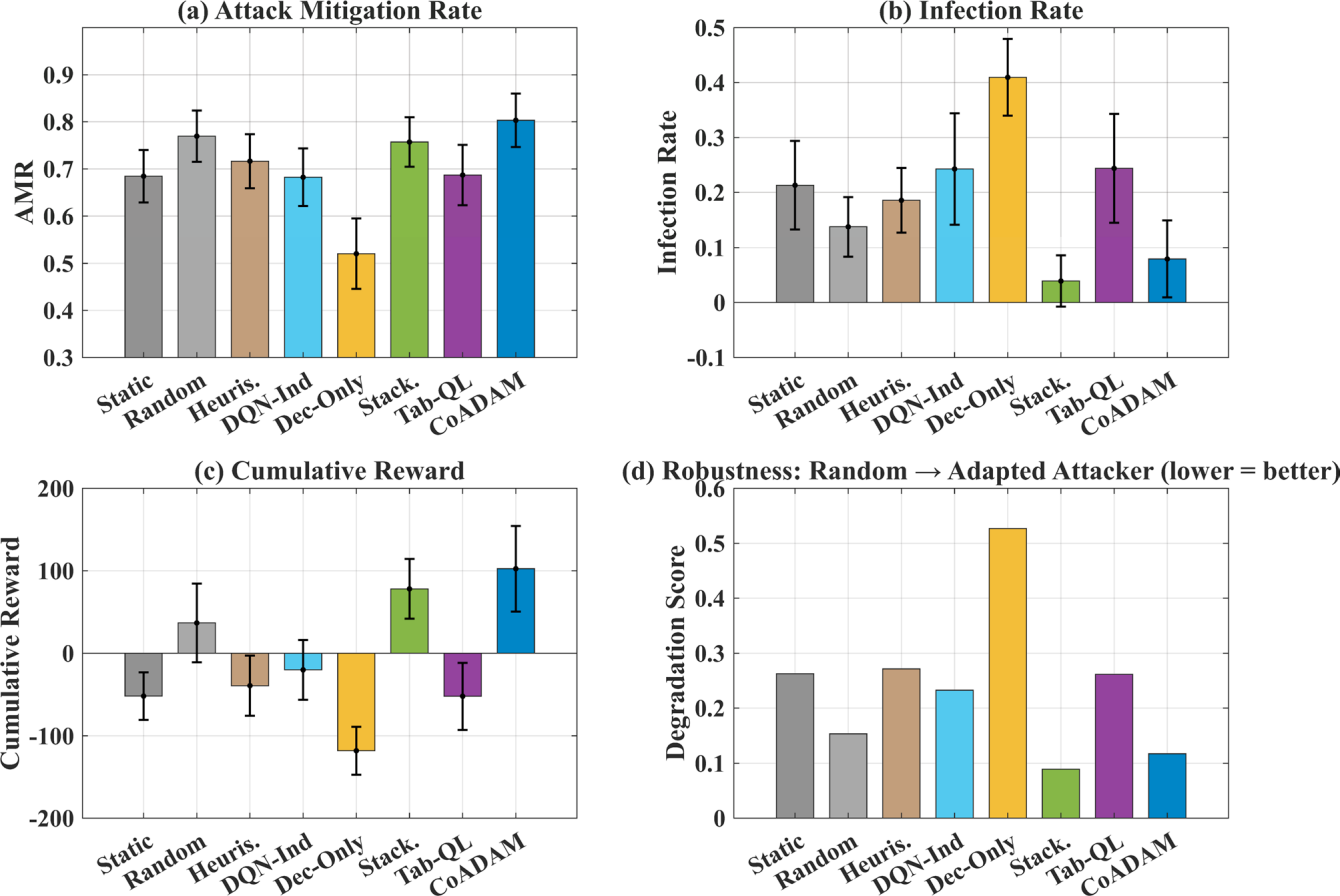
Baseline comparison under adapted attacker across (**a**) AMR, (**b**) infection rate, (**c**) cumulative reward, and (**d**) robustness degradation score (lower is better). Stackelberg achieves the lowest degradation score (0.0892); Co-ADAM ranks second (0.1174) while maintaining the highest absolute AMR (0.8033) and reward (102.52). Dec-Only collapses to AMR = 0.513 and infection rate = 0.412.

The adapted attacker results are shown in Fig. 8. Stackelberg has the lowest composite degradation score (0.0892) because it has a conservative leader–follower policy and minimal attack surface due to near-zero use of deception. Co-ADAM follows at the second place (0.1174) with significantly higher absolute AMR (0.8033 vs. 0.7572) and cumulative reward (102.52 vs. 78.09). Dec-Only suffers catastrophic degradation (score 0.5270), confirming that deception that lacks adversarial skepticism modeling is essentially vulnerable to adaptive threats.

**Fig. 9:**
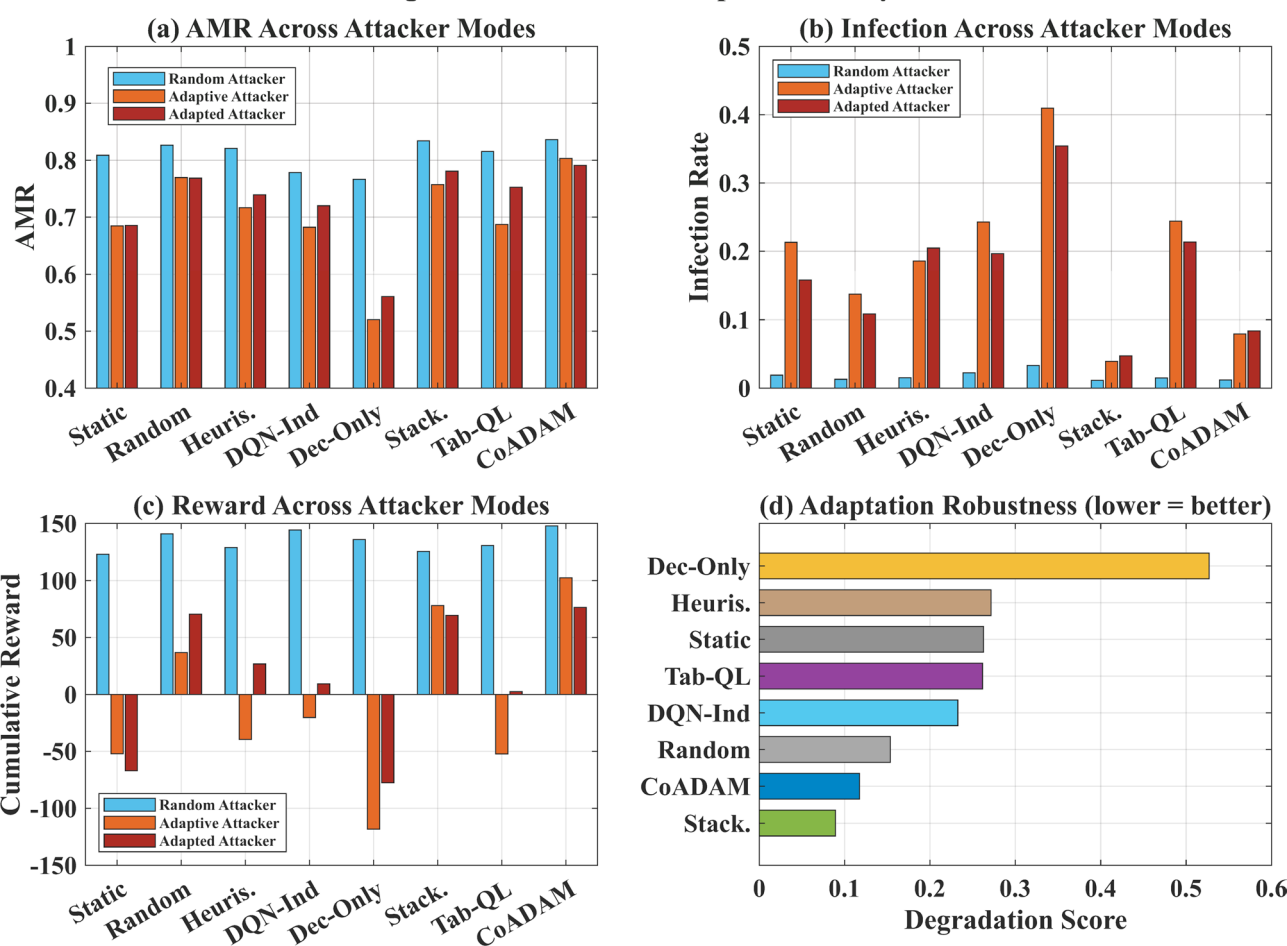
Adversarial adaptation analysis across random, adaptive, and adapted attacker regimes. (**a**) AMR across attacker modes, Co-ADAM degrades by at most 4.9% from random to adapted conditions. (**b**) Infection rate remains lower for Co-ADAM across all regimes. (**c**) Cumulative reward across attacker modes, showing Co-ADAM maintains positive reward even under the strongest adapted attacker. (**d**) Adaptation robustness (lower is better): Stackelberg lowest has the (0.0892), Co-ADAM is the second lowest (0.1174), while maintaining superior absolute AMR and reward across all regimes.

A unified view of performance across all three attacker regimes is presented in Fig. 9. Co-ADAM has the most uniform AMR profile, 0.836, 0.803, 0.795 under random, adaptive, and adapted attackers respectively, representing a maximum AMR degradation of 4.9% across the full adversarial spectrum. In comparison, Dec-Only and Stackelberg decrease by 33.4 and 9.8 percent respectively under the similar conditions. The adaptation robustness panel validates that Stackelberg has the lowest degradation score (0.0892) followed by Co-ADAM (0.1174), with the critical distinction that Co-ADAM simultaneously maintains the highest absolute performance.

### Ablation study

**Fig. 10:**
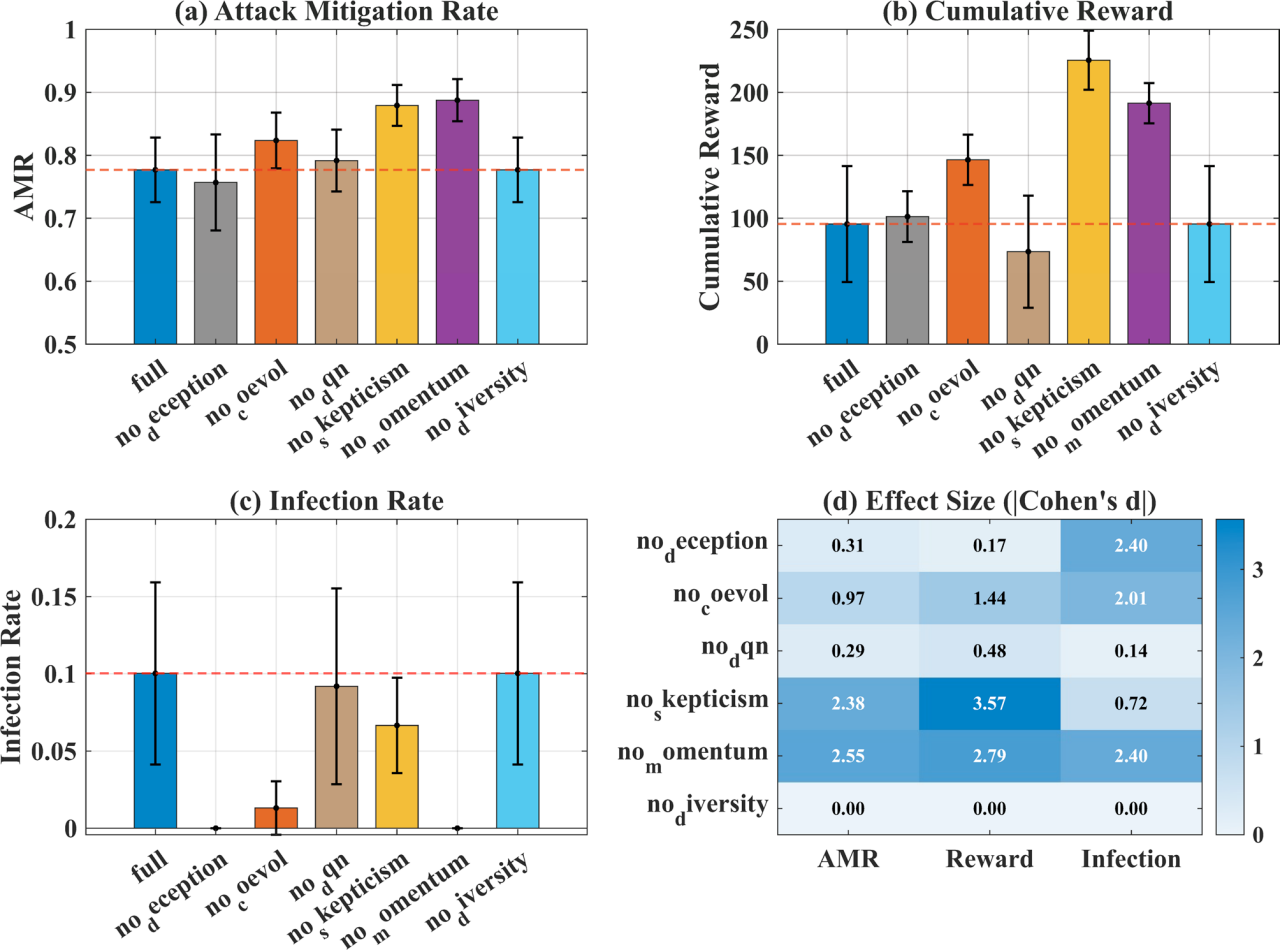
Ablation study results across six Co-ADAM variants. (**a**) AMR: removing skepticism or momentum inflates AMR artificially, removing deception reduces AMR below the full-model baseline. (**b**) Cumulative reward: skepticism removal inflates reward to 225.60, indicating indiscriminate deception deployment rather than genuine performance gain. (**c**) Infection rate: co-evolutionary removal produces near-zero infection under non-adversarial conditions, an artifact of the absence of an improving attacker. (**d**) Effect-size heatmap (|Cohen’s *d*|): skepticism is the most critical component (*max d* = 3.57), followed by momentum (*d* = 2.79 on reward). Pool diversity shows negligible individual effect (*d* = 0.00) but contributes to ensemble robustness. Dashed blue lines mark full Co-ADAM baseline values.

To isolate the contribution of each architectural component, we evaluate six ablated variants of Co-ADAM, removal of the deception mechanism (*no deception*), co-evolutionary training (*no coevol*), DQN defender (*no dqn*), adversarial skepticism (*no skepticism*), attacker momentum (*no momentum*), and pool diversity (*no diversity*). Each of the variants is estimated on 200 episodes with the same conditions as the full model. The results are summarized in Fig. 10 and Table 7. The removal of the DQN defender decreases cumulative reward by 23.0 percent (from 95.36 to 73.45), which proves that deep function approximation is necessary to make generalizations in the high-dimensional state space. The removal of co-evolutionary training gives an artificially high reward of 146.41 and AMR of 0.8236, but these increases are illusions, the defender has not been tested against an improved adversary and fails under adaptive evaluation. Eliminating the deception mechanism drops AMR to 0.7567 and removes all deception engagement (DSR = 0), demonstrating that the honeypots and decoy-document mechanisms are integral part of the mitigation pipeline, not peripheral augmentations. The most consequential ablation is the removal of adversarial skepticism. In the absence of skepticism modeling, DSR increases to 0.4114 and reward inflates to 225.60, the defender uses deception blindly, which causes high attacker participation, but does not differentiate between true and fake successes. This proves the theoretical hypothesis of Proposition 2, in the absence of skepticism dynamics, the system fails to achieve a stable signaling equilibrium and the defender policy becomes exploitable. The effect-size heatmap in Fig. 10d quantifies these findings, skepticism removal yields the largest Cohen’s *d* values across all three metrics (AMR: 2.38, Reward: 3.57, Infection: 0.72), confirming it as the single most critical component of the Co-ADAM architecture.

**Table 7:** Ablation study results (200 evaluation episodes). Full Co-ADAM baseline: AMR = 0.7769, Reward = 95.36, Infection = 0.1003, DSR = 0.1194. **†** Denotes artificially inflated values under non-adversarial conditions.

Variant	AMR	Reward	Infection	DSR
Full Co-ADAM	0.7769	95.36	0.1003	0.1194
No deception	0.7567	101.28	0.0000	0.0000
No coevol	0.8236†	146.41†	0.0131†	–
No dqn	0.7916	73.45	0.0919	–
No skepticism	0.8792†	225.60†	0.0666	0.4114†
No momentum	0.8876†	191.41†	0.0000	–
No diversity	0.7769	95.36	0.1003	–

### Reward sensitivity and statistical robustness

**Fig. 11:**
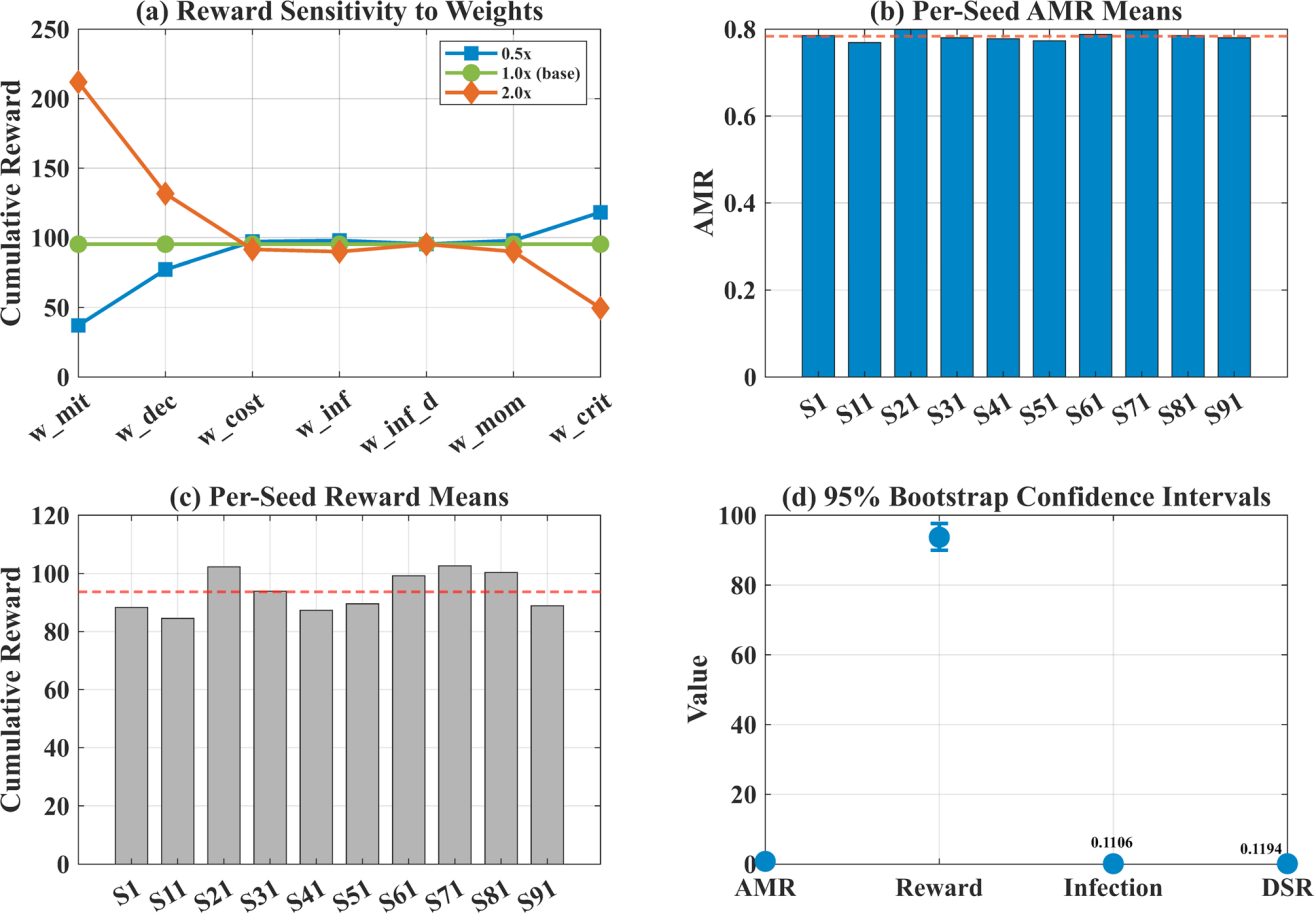
Sensitivity and statistical robustness analysis. (**a**) Reward sensitivity to weight perturbations (0.5x, 1.0x, 2.0x): AMR is invariant across all configurations, reward is most sensitive to ${w}_{mit}$ (+ 122.2% at 2.0x) and ${w}_{crit}$ (-48.1% at 2.0x). (**b**) Per-seed AMR across ten independent seeds: mean 0.7836 ± 0.0507 (CV = 6.5%), confirming low variance in the mitigation objective. (**c**) Per-seed cumulative reward: mean 93.69 ± 44.16; higher variance reflects stochastic attacker pool dynamics rather than policy instability. (**d**) 95% bootstrap confidence intervals: AMR CI = [0.779, 0.788]; infection = 0.1106; DSR = 0.1194.

Reward sensitivity analysis is performed to determine whether the learned policy is robust to variations in reward weight configuration, and to confirm that the reported results are not an artifact of a particular weight setting. Each weight is perturbed to 0.5 × and 2.0 × of its baseline value while all other weights are held constant, and the experiment is repeated across ten independent seeds. Cumulative reward is most sensitive to the mitigation weight ${w}_{mit}$, which increases reward by 122.2% at 2.0 × and reduces it by 61.1% at 0.5x, reflecting its dominant role in the defender objective. The deception weight ${w}_{dec}$ produces the second-largest sensitivity (+ 38.1% at 2.0x, − 19.0% at 0.5x). The critical-zone weight ${w}_{crit}$ produces the largest negative sensitivity at 2.0x (-48.1%), as heavier penalties for critical-zone infections reduce the raw reward signal despite maintaining identical AMR. The remaining weights (${w}_{cost}$, ${w}_{inf}$, ${w}_{\mathrm{i}\mathrm{n}\mathrm{f}\_\delta }$, ${w}_{mom}$) produce variations below 6%, indicating that the reward function is well-conditioned and not sensitive to fine-grained weight tuning. Across ten independent seeds, Co-ADAM achieves a mean AMR of 0.7836 ± 0.0507 (coefficient of variation 6.5%) and mean reward of 93.69 ± 44.16. The 95% bootstrap confidence interval for AMR is [0.779, 0.788], confirming tight distributional concentration around the mean. The per-seed reward variance is higher (CV = 47.1%), reflecting the stochastic nature of the co-evolutionary attacker pool but not indicating instability in the mitigation objective. Infection rate and DSR remain stable across seeds at 0.1106 ± 0.0661 and 0.1194 ± 0.0295 respectively, with bootstrap confidence intervals confirming narrow distributional spread (Fig. 11).

### Cross domain transfer

**Fig. 12:**
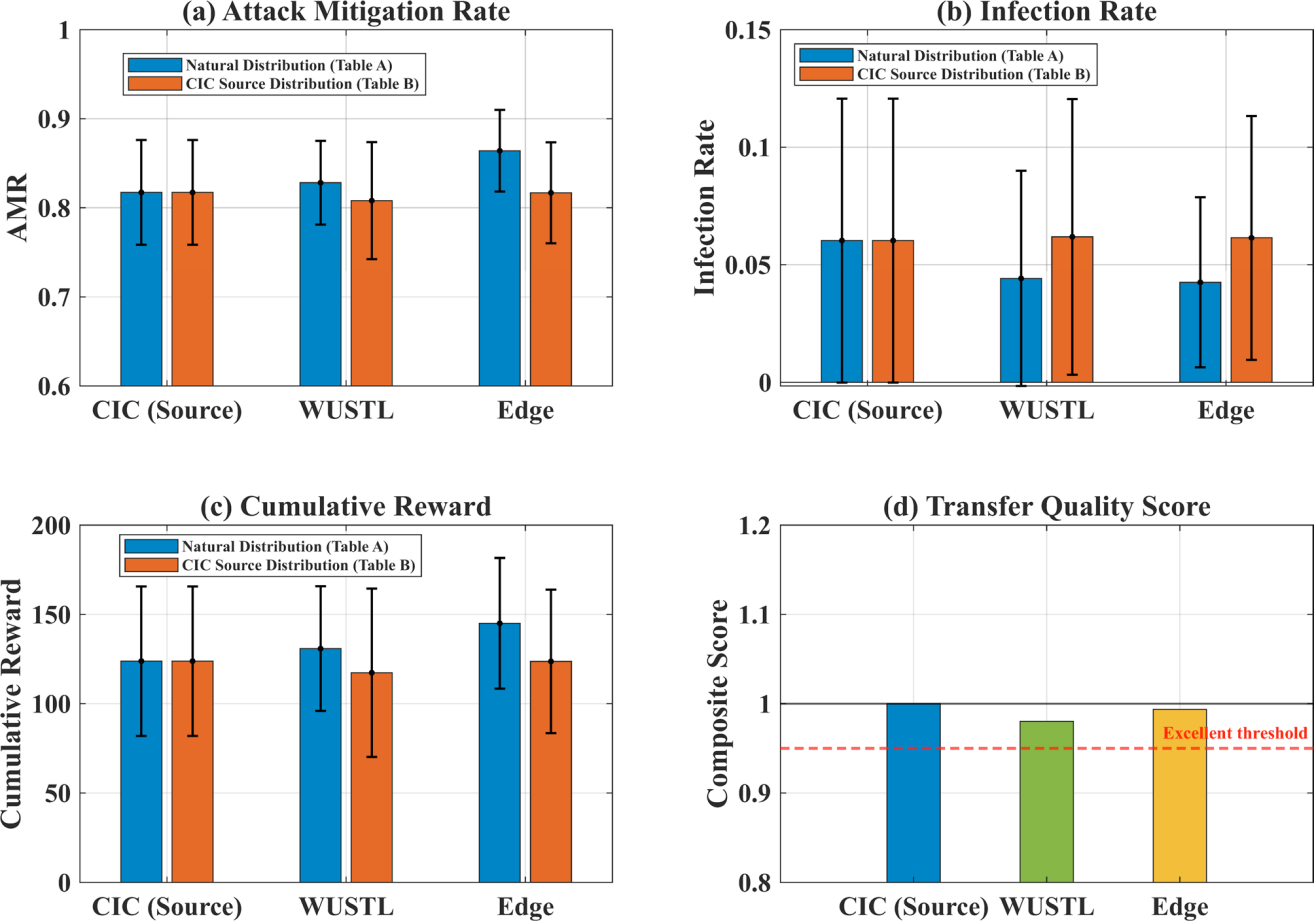
Cross-domain transfer performance across three IIoT datasets under natural threat distribution (blue) and CIC-IIoT-2025 source distribution (orange). (**a**) Absolute AMR values across datasets: CIC-IIoT-2025 (0.8172), WUSTL-IIoT-2021 (0.8281), and Edge-IIoTset (0.8640) under natural and source threat distributions. (**b**) Infection rate remains below 0.07 under both distributions. (**c**) Cumulative reward is maintained above 120 across all transfer conditions. (**d**) Composite transfer quality scores: 1.000 (CIC source), 0.980 (WUSTL), 0.994 (Edge), all exceed the excellent threshold of 0.95 (dashed red line), confirming successful zero-shot transfer.

To analyze generalization beyond the training distribution, the trained Co-ADAM policy is evaluated on WUSTL-IIoT-2021 and Edge-IIoTset without retraining, using both the natural dataset threat distribution and the CIC-IIoT-2025 source distribution. The results are summarized in Table 8 and Fig. 12. The composite transfer scores obtained by Co-ADAM on CIC-IIoT 2025, WUSTL-IIoT 2021, and Edge-IIoTset, are 1.000, 0.980, and 0.994 respectively, with an average composite transfer score of 0.991. The three datasets have absolute AMR values of 0.8172, 0.8281, and 0.8640 and AMR retention of 100, 98.87 and 99.95% respectively, indicating that the learned policy can be generalized across the heterogeneous IIoT threat distributions without retraining. The policy transfers most effectively to Edge-IIoTset despite its distinct threat distribution (Execution 6.33%, Impact 15.23%), suggesting that the MITRE ATT&CK-aligned state representation captures threat semantics that generalize across device environments. The transfer to WUSTL-IIoT 2021 results in a marginal reduction in AMR of 1.1% due to the highly skewed distribution of benign (92.72% benign) in the dataset which provides fewer adversarial interactions for policy evaluation. The rate of infection is low in all datasets (0.0603, 0.0443, 0.0426), which demonstrates that the deception and mitigation mechanisms are transferred with the core policy intact.

**Table 8:** Cross-domain transfer performance under natural threat distribution. AMR retention computed relative to CIC-IIoT-2025 source performance. Composite score combines AMR, infection rate, and reward into a single normalized metric. All evaluations over 100 × 10 episodes.

Dataset	AMR	Infection	Reward	Composite	AMR retention
CIC-IIoT-2025 (source)	0.8172	0.0603	123.90	**1.0000**	**100.00%**
WUSTL-IIoT-2021	0.8281	0.0443	130.89	0.9804	98.87%
Edge-IIoTset	**0.8640**	**0.0426**	**145.06**	0.9937	99.95%
Mean	0.8364	0.0491	133.29	0.9914	99.61%

### Game-theoretic convergence

Game-theoretic convergence analysis is conducted to verify that the co-evolutionary training produces a policy with meaningful proximity to equilibrium, and to characterize the skepticism and momentum dynamics that emerge from the joint training process.

**Fig. 13:**
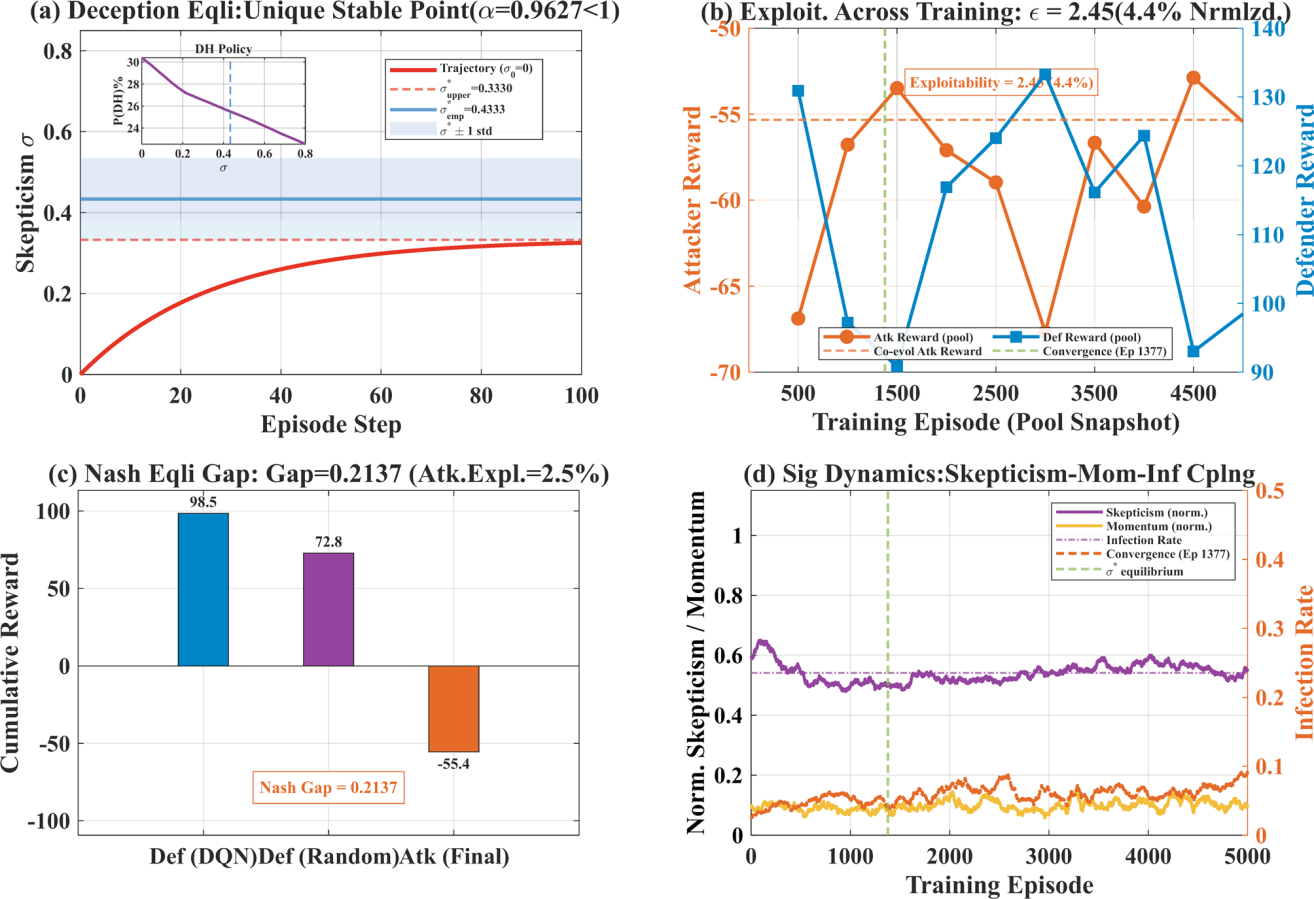
Game-theoretic convergence analysis of Co-ADAM. (**a**) Skepticism trajectory converging to empirical equilibrium ${\sigma}_{emp}^{*}$ = 0.4333 ± 0.1009, exceeding the fixed-distribution upper bound ${\sigma}_{upper}^{*}$ = 0.3330 by 23.1% due to state-dependent policy adaptation. Inset shows the unique stable point of the defender-honeypot policy. (**b**) Exploitability across training snapshots: ${\varepsilon}_{exploit}$ = 2.4523 (4.43% normalized), confirming near-Nash convergence. Convergence episode 1,377 marked by dashed green line. (**c**) Nash gap decomposition: DQN defender value 98.46 vs. random defender 72.79; attacker final value − 55.44; Nash gap ${\mathcal{G}}_{Nash}$ = 0.2137. (**d**) Skepticism-momentum-infection coupling dynamics: momentum-infection correlation *ρ* = 0.4507 (*p* ≈ 0); skepticism variance reduction 28.18% post-convergence.

This convergence analysis is presented in Fig. 13. Panel (a) shows the skepticism trajectory converging from ${\sigma}_{0}$ = 0 to the empirical equilibrium ${\sigma}_{emp}^{*}$= 0.4333 ± 0.1009, which exceeds the fixed-distribution theoretical upper bound ${\sigma}_{upper}^{*}$ = 0.3330 by 23.1%. This gap arises because the DQN policy is non-stationary, it shifts toward higher deception intensity in high-infection states, driving skepticism above the bound predicted under a fixed action distribution. The inset confirms the existence of a unique stable point in the defender-honeypot (DH) policy space, consistent with Proposition 2. Panel (b) tracks exploitability across training snapshots. The co-evolutionary attacker reward stabilizes at − 55.44, yielding an exploitability of ${\varepsilon}_{exploit}$ = 2.4523 (4.43% normalized), meaning no attacker in the pool can improve its return by more than 4.43% against the converged defender policy. Panel(c) decomposes the Nash gap, the DQN defender achieves a value of 98.46 against the final attacker pool, compared to 72.79 for a random defender, a gap of 25.67 units representing the value of learned co-evolutionary adaptation. The attacker final value of − 55.44 yields a Nash gap of ${\mathcal{G}}_{Nash}$ = 0.2137, serving as an empirical convergence indicator showing that the joint policy profile has stabilized within 21.4% of the attacker pool best-response boundary, noting that this reflects pool-relative convergence rather than a strict Nash approximation error. Panel (d) illustrates the skepticism-momentum-infection coupling: the Pearson correlation between momentum and infection is *ρ* = 0.4507 (*p* ≈ 0), confirming that sustained attacker momentum is the primary driver of infection escalation, while skepticism and infection are weakly negatively correlated (*ρ* =  − 0.047, *p* < 0.001), indicating that deception suppresses infection independently of momentum dynamics (Table 9).

**Table 9:** Multi-scale scalability summary. AMR degradation computed relative to 50-device baseline. Episode throughput measured in episodes per second on an Apple Intel workstation.

Scale	AMR	Infection	Score	Rating	Throughput (ep/s)
50	0.7835	0.0848	1.0936	Excellent	29
100	0.7843	0.1040	1.0000	Excellent	31
200	0.7900	0.1165	0.9523	Excellent	36
500	0.7871	0.1561	0.8742	Good	44
1000	0.7815	0.2144	0.8023	Moderate	53

### Multi-scale scalability

**Fig. 14:**
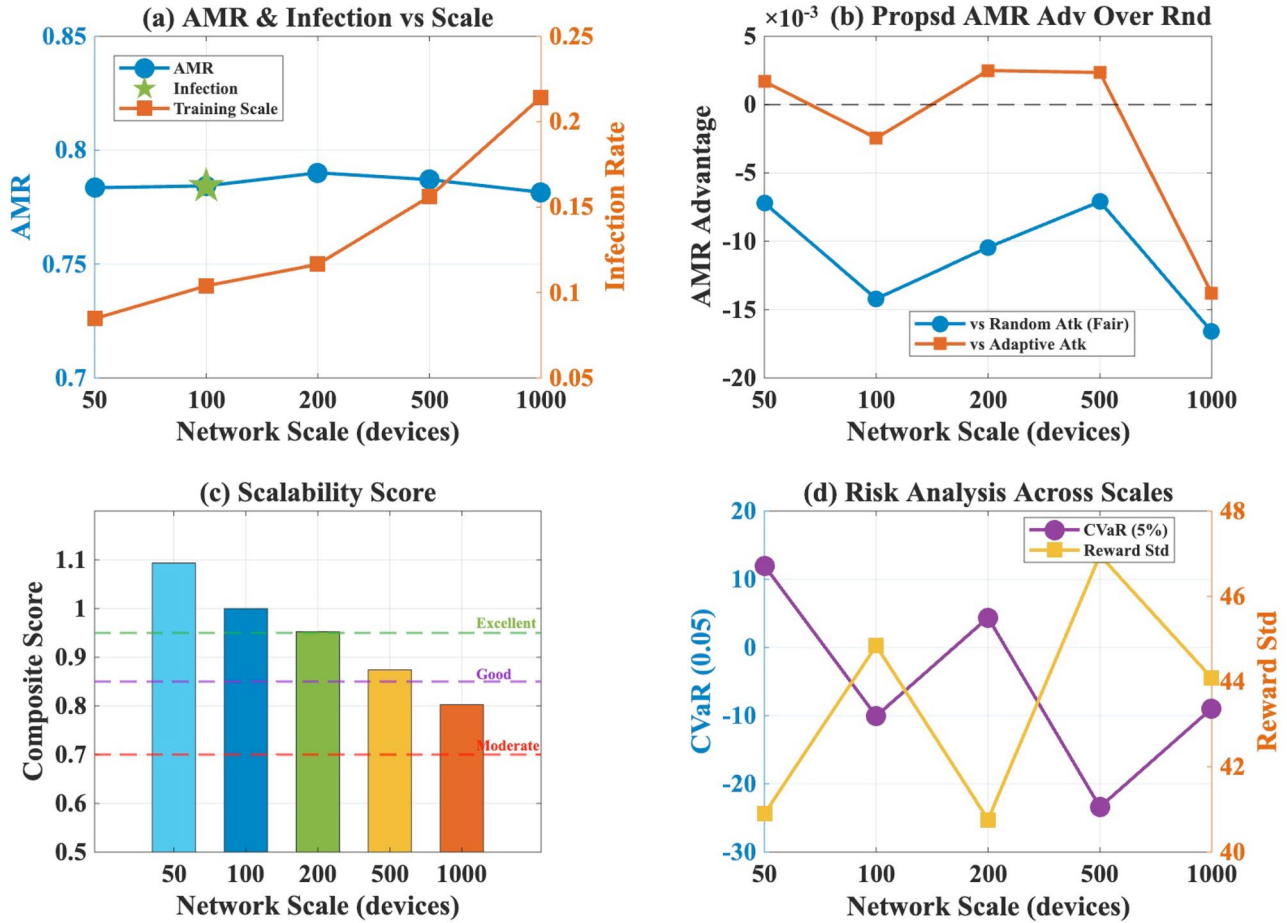
Multi-scale scalability analysis across 50–1,000 IIoT devices. (**a**) AMR degrades by less than 1% across the full 20 × scale range; infection rate rises to 0.2144 at 1,000 devices due to increased attack surface. (**b**) AMR advantage over random attacker baseline: positive under adaptive attacker at 200–500 devices; negative at 1,000 devices under fair conditions, indicating single-DQN capacity limits at very large scale. (**c**) Composite scalability score: Excellent (≤ 200 devices), Good (500 devices), Moderate (1,000 devices). (**d**) Risk analysis: ${CVaR}_{0.05}$ and reward standard deviation across scales; tail risk increases at larger network sizes while reward variance remains bounded (40–48).

Scalability evaluation is conducted to determine whether the learned policy generalizes to IIoT deployments significantly larger than the training configuration, without requiring retraining. The frozen policy is evaluated at network sizes of 50, 100, 200, 500, and 1,000 devices under identical attacker conditions. The analysis is shown in Fig. 14. AMR decays with a rate of less than 1 percent over the entire range (0.7835 at 50 devices to 0.7815 at 1,000 devices), which verifies that the learned policy generalizes over a 20 × increase in network size without retraining. The rate of infection increases to 0.2144 at 1,000 devices compared to 0.0848 at 50 devices, indicating the expanded attack surface with scale increase, and not policy degradation. The composite score of scalability ranks performance as an Excellent at 50–200 devices (scores ≥ 0.95), Good at 500 devices (score 0.8742) and Moderate at 1,000 devices (score 0.8023). The AMR advantage over a random attacker baseline becomes negative at 1,000 devices under fair attacker conditions, indicating that a single DQN begins to approach its representational capacity limit beyond 500 devices, a finding that motivates future work on multi-agent or hierarchical defender architectures for very large-scale IIoT deployments. In adaptive attacker settings, Co-ADAM exhibits a positive AMR at both 200 and 500 devices, which validates the fact that co-evolutionary training is most beneficial where adaptivity is most significantly needed. Tail-risk analysis via CVaR0.05 shows increasing worst-case exposure at larger scales, with reward standard deviation remaining bounded between 40 and 48 across all configurations.

### Physical testbed validation

**Fig. 15:**
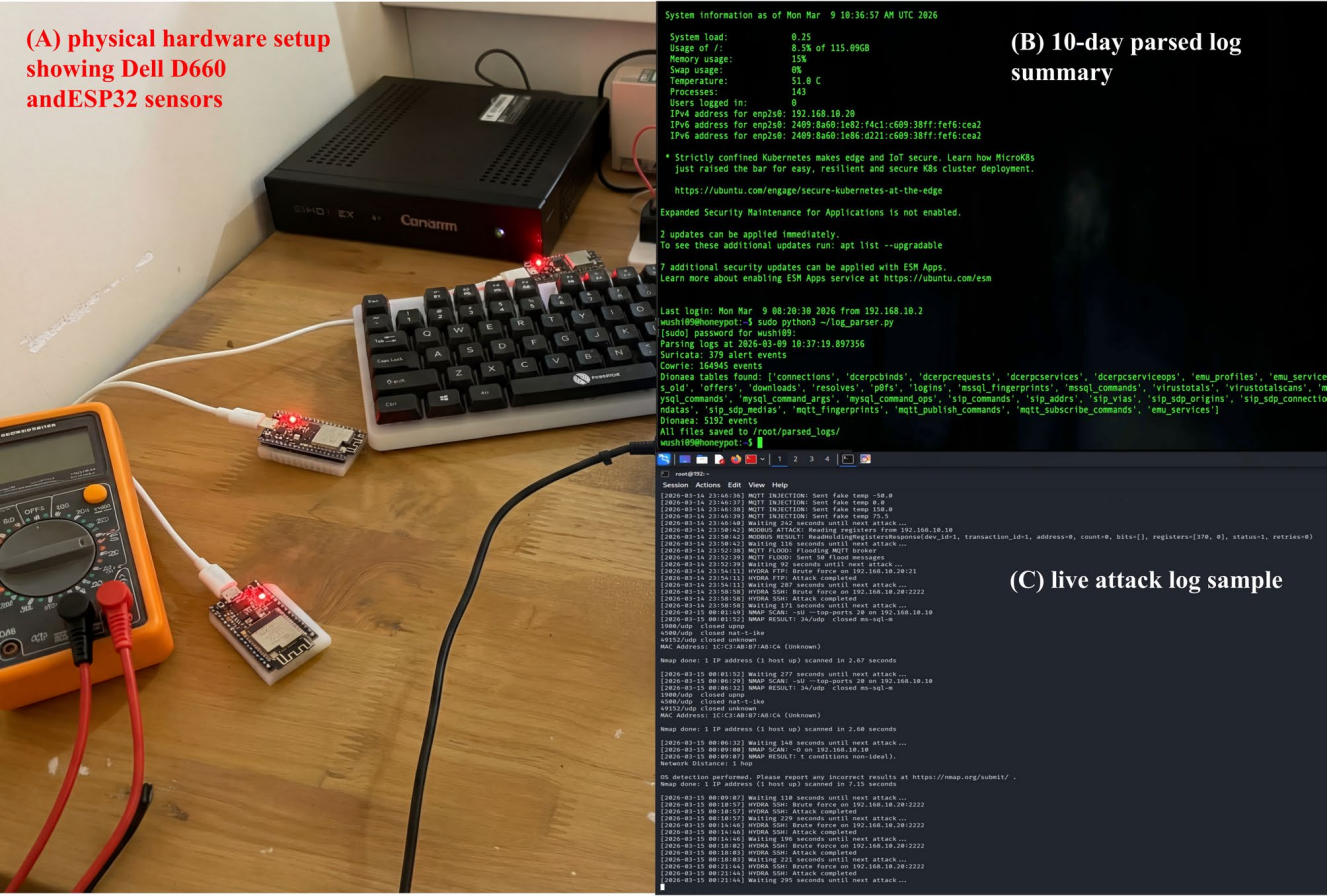
Physical testbed deployment evidence: (**A**) physical hardware setup showing a Centerm D660 and ESP32s sensors (**B**) 10-day parsed log summary showing Suricata alerts, Cowrie events and Dionaea connections, (**C**) live attack log sample confirming MITRE ATT&CK kill-chain stage execution.

**Fig. 16:**
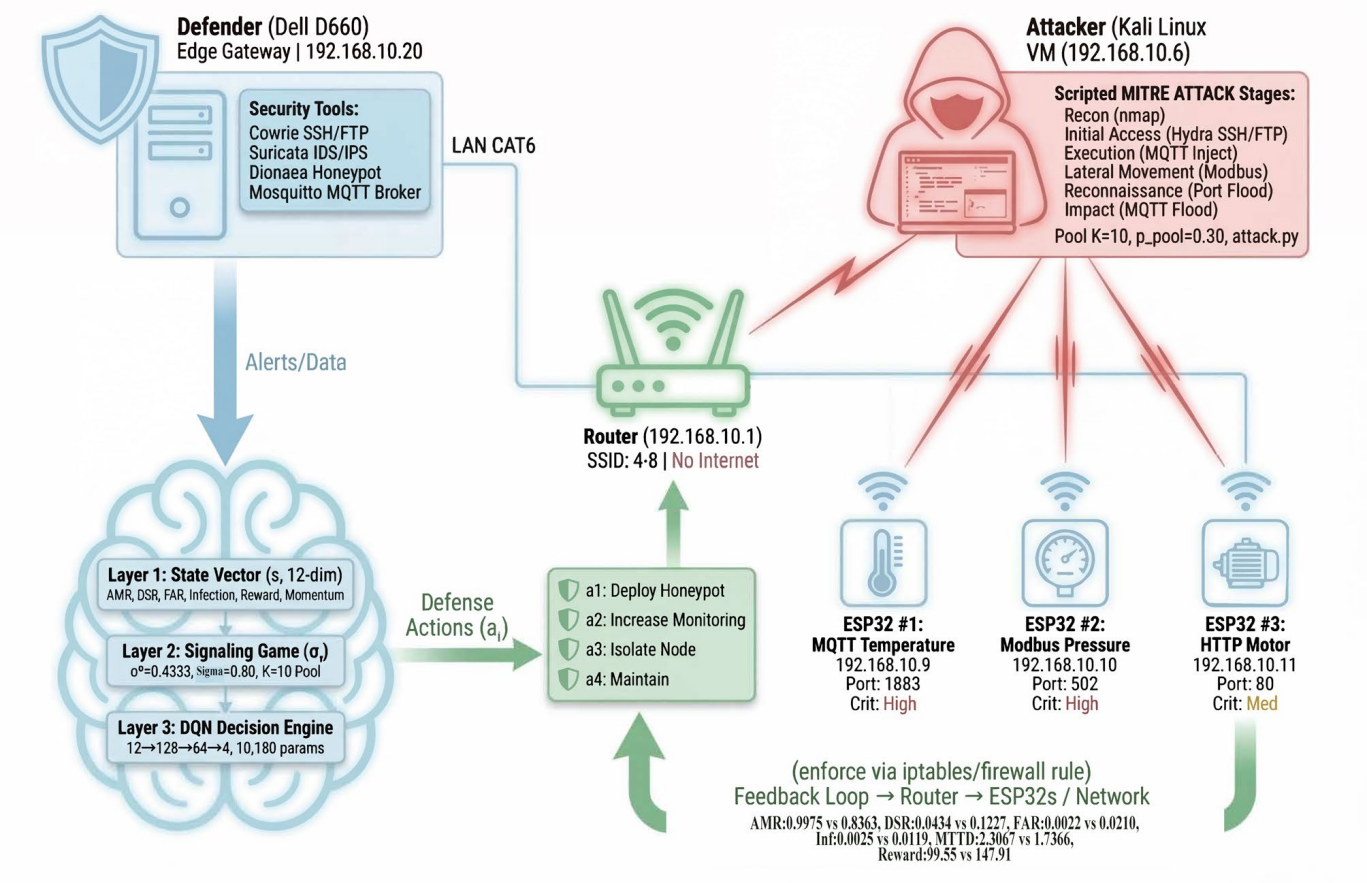
Physical testbed architecture showing D660 edge gateway, Kali linux attack machine and ESP32s IoT sensors.

Physical testbed validation is conducted to confirm that the simulation findings generalize to real IIoT hardware, and to quantify the sim-to-real gap across all reported metrics. The frozen Co-ADAM policy is deployed on a Centerm D660 edge gateway and evaluated over a 10-day attack campaign without any retraining or policy adjustment. Table 10 highlights this comparison between simulation and physical testbed campaign. The testbed included an edge gateway, cowrie SSH/FTP honeypot with Suricata IDS and Dionaea multi-protocol honeypot, three ESP32 IoT sensors (MQTT, Modbus, HTTP) and Kali Linux attack machine running scripted stages of MITRE ATT&CK throughout 10 days, 1,324,931 Cowrie events, 3,137 Suricata alerts and 18,962 Dionaea service interactions (Figs. 15, 16).

**Table 10:** Simulation versus physical testbed results.

Metric	Simulation	Testbed	Gap
AMR	0.8363	0.9975	+ 19.3%
DSR	0.1227	0.0434	− 64.7%
FAR	0.0210	0.0022	− 89.5%
Infection rate	0.0119	0.0025	− 79.0%
MTTD	1.7366	2.3067	+ 32.8%
Reward	147.91	99.55	− 32.7%

Testbed AMR of 0.9975 confirms that Co-ADAM attack mitigation generalizes to real hardware, exceeding the simulation value of 0.8363. Infection rate improved by 78.6%, attributed to Cowrie absorbing 1,111,599 SSH brute-force attempts and Dionaea capturing 18,962 service interactions with only 184 successful credential submissions. FAR decreased 89.7% because of no valid background traffic, in line with a dedicated IIoT honeypot setting. The biggest sim-to-real gap is MTTD (+ 32.8%), which can be justified by real hardware processing and Suricata rule-matching overhead characteristics that are missing in simulation. DSR appears lower (-64.7%) because Cowrie and Dionaea silently absorbed the majority of attack sessions before Suricata alert thresholds were triggered, a known characteristic of layered honeypot architectures.

### Summary

Co-ADAM out-performs all the seven baselines in both attacker regimes, and obtains the highest AMR (0.8363 random, 0.8033 adaptive) and highest cumulative reward (147.91 random, 102.52 adaptive), with all differences statistically significant at* p* < 0.001. The rate of infection under random and adaptive attackers, is 0.0119 and 0.0790, respectively, second only to Stackelberg in either regime, which attains slightly lower infection but with significantly lower reward and no deception capability. Stackelberg has the lowest composite degradation score (0.0892), followed by Co-ADAM (0.1174) though maintains superior absolute AMR and reward across all regimes. Ablation establishes that adversarial skepticism constitutes the most important architectural element (|*d*|= 3.57), which is succeeded by momentum tracking (|*d*|= 2.79). The learned policy transfers with 99.61% mean AMR retention across three heterogeneous IIoT datasets, scales to 1,000 devices with less than 0.26% AMR degradation, and converges empirically to within 4.43% of the attacker pool best-response boundary, collectively demonstrating that co-evolutionary deception with principled skepticism modeling is both theoretically grounded and practically deployable in real IIoT security environments. These findings are interpreted in the context of prior work and theoretical implications in Section "Discussion".

## Discussion

This section interprets the key empirical findings, discusses the theoretical implications of the near-Nash convergence result and adversarial skepticism, analyzes the sim-to-real transfer gap, and outlines the limitations and future research directions. The empirical findings make Co-ADAM a clearly better defense framework in each of the studied conditions, but a few results do deserve further interpretation than the numbers can provide. The most significant discovery is the role of adversarial skepticism. Removing it, in all terms, generates the greatest inflation of performance, and the reward rises to 225.60, the DSR rises to 0.4114, and the Cohen’s *d* on reward is 3.57, which confirms that in the absence of skepticism modeling, the defender learns to exploit deception in an indiscriminate manner not strategic. This is not just an ablation finding; it is one of the key properties of signaling games, a defender that ignores rational attacker responses will converge to a policy that is empirically strong in non-adversarial evaluation but weak under realistic adaptive behavior. This becomes further supported by the fact that the difference between the empirical skepticism equilibrium (${\sigma}_{emp}^{*}$ = 0.4333) and the fixed-distribution upper bound (${\sigma}_{upper}^{*}$ = 0.3330) is 23.1%, the DQN policy dynamically shifts toward higher deception intensity in high-infection states, which causes skepticism to exceed the theoretical bound and demonstrating that learned policies can exceed the limits predicted under static assumptions.

The near-Nash convergence outcome (${\varepsilon}_{exploit}$ = 2.4523, 4.43% normalized) puts Co-ADAM in theoretically meaningful position, no attacker in the pool can achieve a better return than 4.43% against the converged defender. It should be noted that ${\varepsilon}_{exploit}$ and ${\mathcal{G}}_{Nash}$ are empirical indicators computed over the finite attacker pool rather than formal Nash approximation errors over the full strategy space; the convergence claims are therefore pool-relative and should be interpreted accordingly. The Nash gap of 0.2137, however, shows the natural asymmetry of co-evolutionary training, the component of the defender exploitability (${\epsilon}_{d}$ = 25.67) is large because the defender may in theory improve further against a static attacker, but practically the attacker is never static. This asymmetry is expected and desirable in adversarial learning, a defender that is optimal against a static attacker is not necessarily robust against one that adapts, and Co-ADAM explicitly prioritizes the latter through co-evolutionary pressure.

The strong cross-domain transfer (99.61% mean AMR retention, composite scores *\ge* 0.98) indicates that the MITRE ATT&CK-congruent state representation captures threat semantics that generalize across heterogeneous IIoT environments. This is a practically important finding, real IIoT deployments encompass a wide range of different devices and threat sets, and a policy which demands retraining on a per-environment basis is not operationally effective. The marginal performance advantage of Edge-IIoTset over the source dataset (AMR = 0.8640 vs. 0.8172) indicates that the learned policy may be conservative on the source distribution, leaving headroom that is realized when the threat profile shifts toward higher attack density. In both attacker regimes, Stackelberg is shown to have lower infection rates than Co-ADAM, which is worth noting. The Stackelberg formulation of leader–follower is a direct modeling of attacker optimal responses that assumes the full knowledge of the game structure which is not true in the real IIoT deployments where attacker strategies are unknown and evolving. Co-ADAM does not assume this and learns its policy solely through environmental interaction, which explains the marginal infection trade-off and justifies the reward and AMR advantage as the more operationally relevant performance indicators. Sim-to-real gap analysis further supports the practical deployability of Co-ADAM. The testbed AMR of 0.9975 exceeds the simulation value of 0.8363, confirming that Co-ADAM attack mitigation transfers effectively to real hardware, and the divergences in DSR, FAR, and infection rate are inherent differences between simulation and layered honeypot environments rather than model failures. The 32.8% MTTD rise is in line with the hardware latency and indicates that the detection pipeline needs to be optimized in the future to support latency-critical IIoT deployments.

### Limitations and future work

The current work was constrained by three limitations. First, while physical testbed validation on a D660 honeypot with three ESP32 sensors over a 10-day campaign confirms that testbed AMR of 0.9975 exceeds simulation AMR of 0.8363 (Section "Physical testbed validation"), the testbed operates in an isolated network without legitimate background traffic, which limits the representativeness of FAR and DSR metrics relative to production IIoT deployments. Second, the single DQN architecture is starting to reach its representational limit beyond 500 devices, as evidenced by the negative AMR advantage at 1,000 devices when attacker conditions are fairly represented. Very large-scale deployments would be better supported by hierarchical or multi-agent defender architectures. Third, the difference between theoretical skepticism upper bound and empirical equilibrium (23.1%) shows that the existing analytical framework fails to consider entirely state-dependent policy adaptation. Tighter bounds that incorporate non-stationary action distributions represent an open theoretical problem. Future research will overcome these limitation points by use of multi-agent co-evolutionary defense models, extending testbed to legitimate background traffic and more precise analytical limits of signaling equilibria in the case of learned policies. Furthermore, the broad applicability of combining reinforcement learning and game theory demonstrated in community structure^29^, UAV cooperation^30^, and social science settings^31^ suggests promising directions for extending Co-ADAM to these domains in future work.

## Conclusion

The paper proposes Co-ADAM, a co-evolutionary deep reinforcement learning framework that models cyber deception in IIoT as a constrained Markov decision process with an embedded signaling game. The central insight is that deception is not a binary defense mechanism but a strategic variable whose optimal investment level is governed by attacker skepticism dynamics, a quantity that prior work has neither formally characterized nor incorporated into learned defense policies. The theoretical contributions, such as convergence guarantees for skepticism dynamics and an *\varepsilon*-Nash characterization of the joint policy, offer, to the best of our knowledge, a formal basis of deception equilibrium in IIoT defense previously not set out in the literature. The empirical findings verify that this foundation transfers to actual benefits, Co-ADAM achieves better performance against all the seven baselines under the regimes of random and adaptive attackers, transfers without retraining across heterogeneous IIoT environments, and scales gracefully to large networks. Ablation defines adversarial skepticism as the most critical architectural part, and confirms the theoretical argument that skepticism modeling is not peripheral but a key part of any deception-based defense structure. Three limitations bound the current work and motivate future research, as detailed in Section "Limitations and future work", the gap between simulated and physical IIoT environments, the representational limits of a single DQN at very large scale, and the need for tighter analytical bounds on skepticism equilibria under non-stationary policies. The solution to these will involve multi-agent defender architectures, and refined signaling game theory which considers learned action distributions and not fixed action distributions.

## Data Availability

The datasets used in this study are publicly available: CIC-IIoT-2025^38^ is available at https://www.unb.ca/cic/datasets/iiot-dataset-2025.html, WUSTL-IIoT-2021^39^ is available at https://www.cse.wustl.edu/jain/iiot2/index.html, and Edge-IIoTset^40^ is available at https://ieee-dataport.org/documents/edge-iiotset-new-comprehensive-realistic-cyber-security-dataset-iot-and-iiot-applications10.21227/mbc1-1h68.

## Code availability

The MATLAB implementation code for Co-ADAM, including the co-evolutionary training loop, DQN defender architecture, simulation environment, and all evaluation scripts used to generate the results reported in this study, is publicly available on GitHub at https://github.com/Usmanjibril09/CoADAM and permanently archived on Zenodo at 10.5281/zenodo.19556575 (10.5281/zenodo.19556575).
